# Therapeutic targets and interventional strategies in COVID-19: mechanisms and clinical studies

**DOI:** 10.1038/s41392-021-00733-x

**Published:** 2021-08-26

**Authors:** Yu-Wen Zhou, Yao Xie, Lian-Sha Tang, Dan Pu, Ya-Juan Zhu, Ji-Yan Liu, Xue-Lei Ma

**Affiliations:** 1 Department of Biotherapy, Cancer Center, West China Hospital, Sichuan University, Chengdu, Sichuan Province China; 2 Key Laboratory of Biotherapy, Cancer Center, West China Hospital, Sichuan University, Chengdu, Sichuan Province China; 3 Department of Dermatovenerology, West China Hospital, Sichuan University, Chengdu, Sichuan Province China; 4 Lung Cancer Center, West China Hospital, Sichuan University, Chengdu, Sichuan Province China

**Keywords:** Infectious diseases, Infection

## Abstract

Owing to the limitations of the present efforts on drug discovery against severe acute respiratory syndrome coronavirus 2 (SARS-CoV-2) and the lack of the understanding of the biological regulation mechanisms underlying COVID-19, alternative or novel therapeutic targets for COVID-19 treatment are still urgently required. SARS-CoV-2 infection and immunity dysfunction are the two main courses driving the pathogenesis of COVID-19. Both the virus and host factors are potential targets for antiviral therapy. Hence, in this study, the current therapeutic strategies of COVID-19 have been classified into “target virus” and “target host” categories. Repurposing drugs, emerging approaches, and promising potential targets are the implementations of the above two strategies. First, a comprehensive review of the highly acclaimed old drugs was performed according to evidence-based medicine to provide recommendations for clinicians. Additionally, their unavailability in the fight against COVID-19 was analyzed. Next, a profound analysis of the emerging approaches was conducted, particularly all licensed vaccines and monoclonal antibodies (mAbs) enrolled in clinical trials against primary SARS-CoV-2 and mutant strains. Furthermore, the pros and cons of the present licensed vaccines were compared from different perspectives. Finally, the most promising potential targets were reviewed, and the update of the progress of treatments has been summarized based on these reviews.

## Introduction

The novel coronavirus disease 2019 (COVID-19) caused by severe acute respiratory syndrome coronavirus 2 (SARS-CoV-2) was first reported in Wuhan, China, in December 2019 and has rapidly become a pandemic.^1^ SARS-CoV-2 has a long incubation period of up to 33 days (in some studies, incubation period of >14 days was registered in >5% of patients with traced contacts)^2^ and a rapid transmission speed, faster than those of other coronaviruses, including SARS-CoV and the Middle East respiratory syndrome (MERS)-CoV. Moreover, asymptomatic carriers may also spread the virus.^3–5^ Most patients infected with SARS-CoV-2 exhibit mild-to-moderate symptoms; however, approximately 15% progress to severe pneumonia^4^ and approximately 5% eventually develop acute respiratory distress syndrome (ARDS), septic shock, multiple organ failure, and even death.^4,6,7^ Owing to the abovementioned characteristics, as of June 1, 2021, COVID-19 spread to >200 countries leading to >170,000,000 identified cases with 3,782,490 confirmed deaths.^8^ The pandemic has increased the susceptibility of humans to microbial pathogens and has revealed the gaps in our therapeutic arsenal; scientists are working at unprecedented speed to understand the disease and to find a cure.

**Fig. 1:**
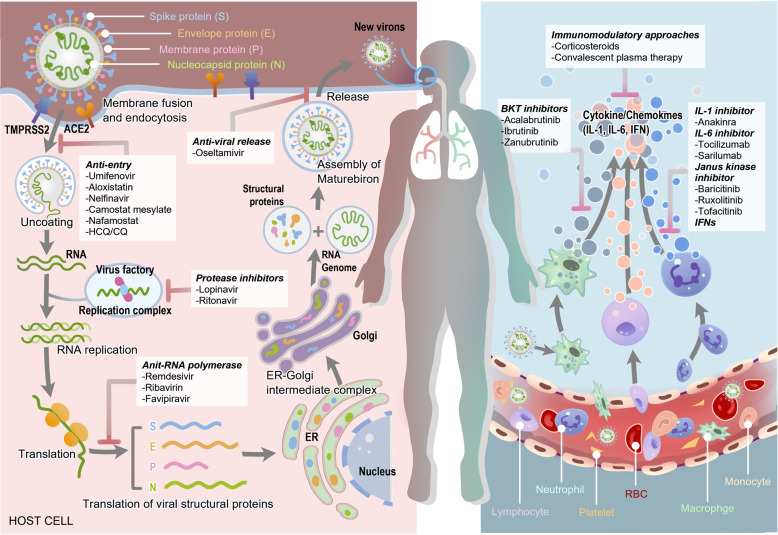
The overview diagram of SARS-CoV-19 invasion and the response of host immune system. The drugs and their corresponding targets are also shown in the diagram

Currently, two main courses are believed to drive the pathogenesis of COVID-19. In the early stage of infection progression, it is primarily driven by the identification, fusion, entry, and replication of SARS-CoV-2, also called as the replication cycle, which is mainly modulated by viral proteins. In the late stage of infection progression, it is driven by a tremendous inflammatory/immune response to SARS-CoV-2 that results in tissue damage. Thus, both the proteins of the virus and host factors are essential for the pathogenesis of COVID-19 and are promising potential targets for antiviral therapy (Fig. 1).

**Fig. 2:**
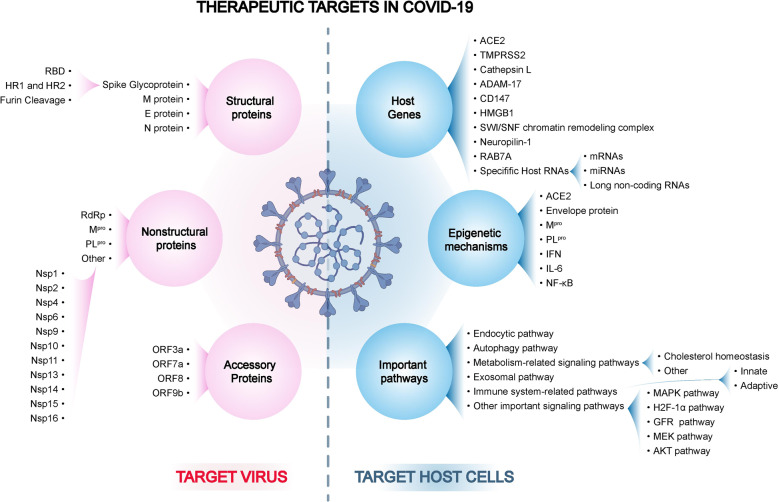
The overview diagram of all therapeutic targets in COVID-19

In this review, based on the above described understanding of the pathogenesis of COVID-19, the therapeutic targets and interventions of COVID-19 have been classified into “target virus” and “target host” categories. A comprehensive analysis of the therapeutic targets has been conducted based on the viral and host factors, occurring at the levels of DNA, RNA, and proteins, involving both classic and novel important signaling pathways and even comprising the promising epigenetic mechanisms, which would contribute to SARS-CoV-2 infection (Fig. 2). Furthermore, a profound analysis has been performed on the highly acclaimed current therapeutic strategies of COVID-19, both based on “target virus” and “target host” categories. Because drugs are being repurposed, emerging approaches and promising potential targets are the implementations of the above two strategies. First, a comprehensive review of the highly acclaimed old drugs was performed according to evidence-based medicine, and the mechanism, potential targets, and already shown clinical data of these drugs were summarized to prepare guidelines for repurposing drugs. Additionally, their unavailability in fighting COVID-19 has been analyzed and summarized. Next, a profound analysis of the emerging drugs has been conducted, particularly including all licensed vaccines and monoclonal antibodies (mAbs). Furthermore, pros and cons of the present licensed vaccines have been compared from different perspectives. Regarding mAbs, the efficacy, adverse events, and administrations of these non-negligible treatments in the management of SARS-CoV-2 have been analyzed. Current vaccines and mAbs have demonstrated efficacy against COVID-19. However, increasing number of mutations emerged worldwide, and these variants pose a significant challenge to current treatments. Thus, the most popular mutations have been summarized, and the efficacy of current licensed vaccines and mAbs against these variants has been reviewed. Finally, the most promising potential targets were reviewed, and preclinical novel drugs were enumerated based on them.

## Pathogenic mechanism

As mentioned above, two main courses between virus and host are thought to drive the pathogenesis of COVID-19: the so-called replication cycle of SARS-CoV-2 and the tremendous inflammatory/immune response to the virus. The fierce virus–host interactions could cause damage to tissues and organs, resulting in severe COVID-19.

**Fig. 3:**
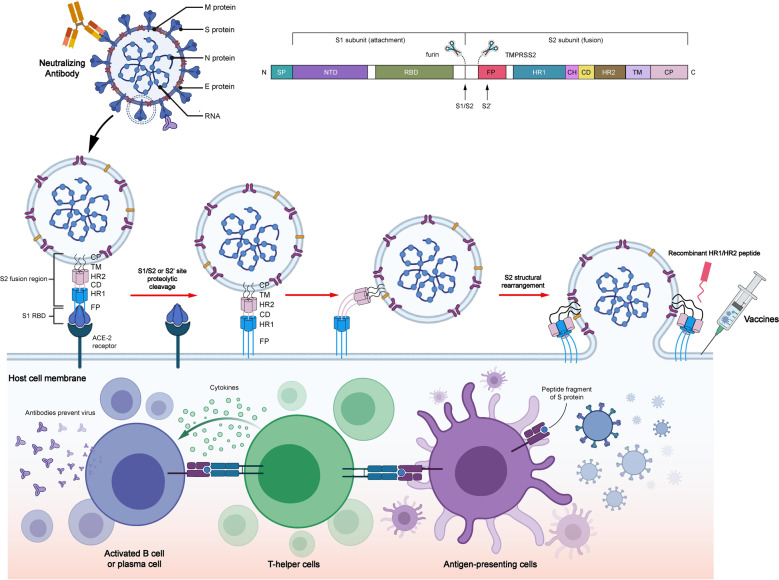
Structure of SARS-CoV-2, spike (S) protein-mediated membrane fusion, and potential therapy against the spike protein. SARS-CoV-2 comprises four structural proteins: S, M, E, and N proteins. Specifically, S protein is composed of two functional subunits, S1 subunit for attachment and S2 subunit for fusion. S1 subunit is composed of NTD and CTD. S1 subunit exerts its effects primarily through RBD in CTD. S2 subunit is made up of FP, a helix–turn–helix structure formed by HR1 and HR2 around a CH, CD, TM, and CT. SARS-CoV-2 is recognized by the binding of RBD and ACE2. Next, the S protein could be hydrolyzed by host proteases at the cleavage spots of S1/S2 (furin) and S2 (TMPRSS2). Then the conformation of S protein is irreversibly changed to further activate the release of the FP structural constraints. S2 subunit is folded to form antiparallel 6-HB by three HR2 segments folding into the grooves on the surface of the HR1 inner core, thereby resulting in the lipid membrane fusion of the virus and the host. Three drugs could fight with S protein containing vaccines and nAbs against S protein and recombinant HR1/HR2 peptides against 6-HB formation. Vaccines against S protein play their role via antigen presentation, cytokine stimulation, and antibody production, whereas nAbs directly bind to S protein to fight with it

During the early stage of infection, the structural integrity and normal functions of virus-related proteins are vital for the virus replication cycle. The structural proteins of SARS-CoV-2 mainly comprise spike (S), membrane (M), envelope (E), and nucleocapsid (N) proteins. Among these, S, M, and E proteins are embedded in the envelope of viral surface, whereas N protein is located in the core of ribonucleoprotein to form the capsid outside.^9,10^ S protein exists as a homotrimer in the virion envelope and contains membrane-distal S1 and membrane-proximal S2 subunits.^11,12^ S protein is associated with the process of virus entry by receptor recognition and fusion mediation. M and E proteins help in the assembly and production of the virion. N protein binds with viral genome and contributes to the virus release. SARS-CoV-2 initiates its invasion after the virus entry into the nasopharynx mucosa. Once the receptor-binding domain (RBD) of S1 subunit directly binds with the angiotensin-converting enzyme-2 (ACE2) of the epithelial cells in the nasopharynx, S1 subunit dissociates, and meanwhile, the spring-loaded S2 subunit refolds, which is conducive for membrane fusion.^13,14^ Notably, the activation of the S protein RBD requires the cleavage of polybasic S1/S2 or S2’ site on the host cell surface by the host proteases, including endosomal cathepsin L (CatL) or transmembrane protease serine 2 (TMPRSS2),^15,16^ followed by which the S protein experiences conformation change to facilitate membrane fusion between the virus and host cell. Therefore, receptor binding and proteolytic activation are two primary processes of virus entry. The higher combination affinity of ACE2 with RBD in SARS-CoV-2 promotes virus entry (Fig. 3).

The biological events that subsequently occur include replication, assembly, and release of virus. The protease of the virus (PLpro) is required to form a proper functional replicase complex and promote viral spread. After the viral genome enters the host cell cytoplasm, it gets translated into replicase proteins (open reading frame 1a/1b (ORF1a/1b)), subsequently undergoing cleavage to form individual nonstructural proteins (Nsps) by PLpro, resulting in the formation of RNA-dependent RNA polymerase (RdRp).^17^ The endoplasmic reticulum (ER) is rearranged by the replicase to form double-membrane vesicles, which are involved in the regulation of replication and transcription of virus (subgenomic RNA (sgRNA)). The transcription of sgRNA results in the formation of structural and accessory proteins. The sgRNAs are inserted into the ER and then moved to the ER–Golgi intermediate compartment for viral budding. Ultimately, the genome enveloped in the N protein assembles to incorporate new virions, which are transported in the vesicle and secreted from the membrane through exocytosis.^18^ Newly encapsulated virus invades other cells and infiltrates body organs owing to blood flowing from the nasal, oral, pulmonary, and the predominant infective body site,^19^ leading to multiple organ impairments in the disease development.^20^

**Fig. 4:**
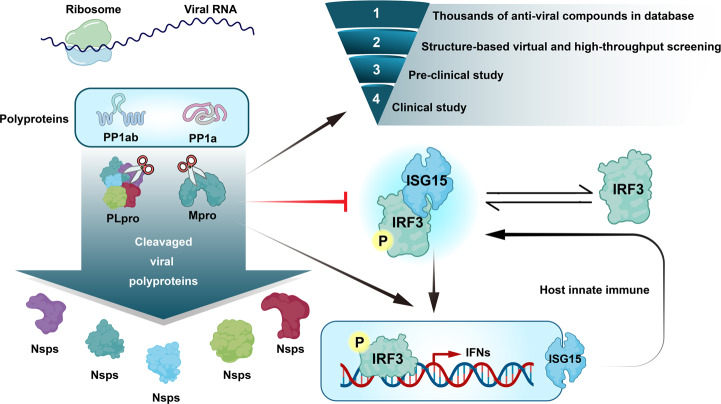
Viral RNA is transcribed to become polyproteins. M_pro_ and PL_pro_ function as a knife, cutting the polyproteins translated from the viral RNA and forming functional viral proteins (Nsp1-16). SCoV2-PL_pro_ also cleaves the ubiquitin-like interferon-stimulated gene 15 protein (ISG15) and reduces type I interferon to further affect host immune response. The development of anti-SARS-CoV-2 drug would undergo four steps: (1) screening thousands of antiviral compounds in database; (2) structure-based viral selecting; (3) preclinical study; and (4) clinical study

Furthermore, the invasive virus and attacked cells strongly trigger uncontrolled “cytokine storm” with hyperinflammatory cytokines, including interleukin (IL)-6, tumor necrosis factor-alpha (TNF-α), and IL-1b.^21,22^ Several studies have also demonstrated the important roles of SARS-CoV-2 viral proteins in the innate and adaptive immunity. Innate immunity is primarily known as the first line to resist foreign agents. This system is rapid, evolutionary, and nonspecific.^23^ Phagocytic leukocytes, epithelial cells, and soluble immune mediators fundamentally comprise the lung innate immune system. When S protein binds with ACE2, the innate immune reaction may get activated via the stimulating nuclear factor κB (NF-κB) cascade in epithelial cells, monocytes, and macrophages.^24^ Then SARS-CoV-2 escapes the host antiviral defenses by employing immune blunting or delay, allowing either rapid replication or by promoting inflammatory reaction.^25,26^ In reverse, several innate immune-associated proteins are targeted by coronavirus proteins. PLpro participates in cleaving host proteins as an evasion mechanism against antiviral immune responses.^27–29^ SARS-CoV-2 distinctively interacts with the amino-terminal ubiquitin-like domain of the ubiquitin-like interferon (IFN)-stimulated gene 15 (ISG15), an important innate immune regulator of host cell. Moreover, preferential cleavage of ISG15 by PL_pro_ may attenuate type I IFN-signaling pathway, an essential component in antiviral response, and IFN responsive factor 3 (IRF3)^30^ (Fig. 4). Other proteins of SARS-CoV-2, including structural protein called N protein and accessory proteins called ORF6 and ORF8, were also demonstrated to be potential inhibitors of type I IFN pathway. Moreover, a clinical study demonstrated the absence of detectable type I IFN in patients with COVID-19.^31^ Apart from NOD-, LRR-, and pyrin domain-containing protein 3 (NLRP3), inflammasome also attracted much attention in the innate immunity response caused by SARS-CoV-2. The binding of S protein and the ACE2 receptor can activate the NLRP3 inflammasome, resulting in pyroptosis.^32^ Subsequently, host cells may die from pyroptosis, after which the pyroptotic epithelial cells can release a large number of virions, which is important for efficient dissemination of SARS-CoV-2 and is also referred to as damage-associated molecular patterns (DAMPs).^33^ The DAMPs trigger multiple signaling pathways, including retinoic acid-inducible gene I and mitochondrial antiviral signaling (MAVS)^34^ and autophagy,^31^ thereby finally inducing the transactivating activities of NF-κB and IRF3 and further producing type I IFN and proinflammatory inhibitors.^23^ Additionally, because the E protein of SARS-CoV allows calcium (Ca^2+^) transport, changes in the Ca^2+^ level in the cytosol would trigger NLRP3 inflammasome pathways.^35^ Owing to the structural similarity between SARS-CoV-2 and SARS-CoV, a hypothesis that the E protein of SARS-CoV-2 regulates the NLRP3 signaling pathway has been proposed.^36^ Furthermore, several coronavirus accessory proteins affecting the function of NLRP3 inflammasome, including ORF3a, have been identified to be involved in the NLRP3 inflammation activation.^36^ These findings need to be experimentally validated further both at basic and clinical levels.

Consistently, the important roles of SARS-CoV-2 viral proteins in the adaptive immunity were also demonstrated. Adaptive immune system can develop protective immunity by responding to pathogens in an antigen-specific manner. There are mainly two kinds of immune cells that comprise the adaptive immune system: B cells and T cells. In vitro, peripheral blood mononuclear cells can be stimulated with peptide pools derived from individual N, M, or S proteins. It has been well established that CD4^+^ and CD8^+^ T cells specific for the peptide pools derived from N, M, and S SARS-CoV-2 proteins are detected in the blood of patients with COVID-19.^37^ M protein-reactive CD4^+^ T cells are the most polyfunctional with increased frequencies of IFN-γ, IL-2, and TNF-α, followed by S protein- and finally N protein-reactive CD4^+^ T cells. Although CD8^+^ T cells were characterized by the production of IFN-γ, the concentration of CD8^+^ T cells was lower than that of CD4^+^ T cells.^38^ Another clinical study found that the level of IFN-γ in response to N or S proteins was higher in patients with mild infection than in severe cases.^39^ Clinical factors, including age and sex, were also associated with CD8^+^ T cell response and COVID-19 prognosis.^40,41^ In patients with severe COVID-19, lung-infiltrating CD8^+^ T cells showed T cell exhausted status with upregulated PD-1 and Tim-3 markers.^42^ Moreover, in patients with mild COVID-19 having CD8^+^ T cells “exhausted” profile, SARS-CoV-2-reactive cells increased in frequency and presented with lower inflammatory characteristics and cytotoxicity. In contrast, in patients with severe disease with CD8^+^ T cell “non-exhausted” profile, SARS-CoV-2-reactive cells showed the stimulation of prosurvival NF-κB and anti-apoptotic pathways. Cumulatively, patients with severe COVID-19 showed robust CD8^+^ T cell memory responses.^43^ These results may highlight that CD4^+^ T cells play a role in the pathogenesis of COVID-19, whereas CD8^+^ T cells are beneficial. Regarding antibody responses, the RBD domain of the SARS-CoV-2 S protein is the primary target of these viral-neutralizing antibodies (nAbs).^44^ Immunoglobulin G (IgG) and IgA were detected in almost all COVID-19 cases, and the positive detected rate of IgM was lower than that of IgG and IgA.^40,45^ The level of IgG, IgM, and IgA titers was consistent with RBD Ig.^40^ Moreover, multiple studies further measured functional antibodies, and the nAbs were almost detected in all subjects.^40,46^ Of note, the titer of nAb was associated with RBD IgG and IgA^40^; these findings further confirmed that RBD is the primary target of nAbs in SARS-CoV-2 infection. The fierce virus–host interactions could cause damage to tissues and organs, resulting in severe COVID-19. Moreover, increasing number of mutations emerged worldwide.

## Target virus

### Antientry

#### Repurposing drugs

Entry is the first step for SARS-CoV-2 to invade host cells. Structural proteins play an important role in this process. As mentioned above, the structural proteins of SARS-CoV-2 mainly comprise S, M, E, and N proteins. Therapeutic strategies are designed to target key elements of structural proteins to inhibit viral entry. Several drugs were considered to have antientry effect and were repurposed in COVID-19.

Umifenovir, also called Arbidol, is a small indole-derivative molecule approved for the prevention and treatment of influenza and other viral infections in the respiratory system in Russia and China. Umifenovir could stabilize the membrane and/or mask the vital residues in receptor recognition^47,48^, thus impairing the attachment of the virus to the plasma membrane. This might impact viral entry.^48^ Some studies have demonstrated favorable clinical response with umifenovir plus lopinavir/ritonavir.^49^ Nojomi et al. have reported that umifenovir showed significant clinical and laboratory improvements, including peripheral oxygen saturation, intensive care unit (ICU) admissions, duration of hospitalization, chest cytoplasmic tail (CT) involvements, white blood cell, and erythrocyte sedimentation rate level, compared with lopinavir/ritonavir.^50^ However, a meta-analysis that included 12 clinical trials and 1052 patients showed no evidence to improve COVID-19 outcomes.^51^ Nelfinavir (Viracept), a kind of protease inhibitor, has been used as an antiretroviral drug in human immunodeficiency virus (HIV) treatment.^52^ Recent experiments have suggested that nelfinavir inhibits S-n- and S-o-mediated cell fusion resulted from SARS-CoV-2 S glycoprotein, thus inhibiting membrane fusion.^53,54^ However, no clinical data are available for nelfinavir.

Chloroquine (CQ) is an antimalarial drug, and hydroxychloroquine (HCQ) is a CQ analog used in treating autoimmune diseases, including systemic lupus erythematosus and rheumatoid arthritis. HCQ could increase the endosomal pH, thus inhibiting the fusion of SARS-CoV-2 and the host cell membranes.^55,56^ Additionally, CQ may interfere with the binding of SARS-CoV to the cell membrane by inhibiting the glycosylation of cellular ACE2 receptor.^57^ An in vitro experiment also suggested an immunomodulatory effect of CQ and HCQ.^58^ Therefore, the efficacy and safety of CQ and HCQ for COVID-19 treatment have been assessed in multiple clinical trials. Unfortunately, compared with the usual standard of care, HCQ did not decrease the 28-day mortality but increased the length of hospital stay and risk of intervention of invasive mechanical ventilation or death.^59^ Therefore, based on the existing evidence, HCQ did not improve the clinical outcomes in hospitalized patients with mild-to-moderate COVID-19, but more adverse events occurred compared with standard care.^60^ Moreover, HCQ with azithromycin showed no benefit for HCQ among hospitalized patients with COVID-19 in retrospective observational studies.^61,62^ In June 2020, Food and Drug Administration (FDA) revoked the emergency use authorization (EUA) of CQ and HCQ in treating certain hospitalized patients with COVID-19^63^ because FDA suggested that CQ and HCQ are unlikely to be effective in COVID-19 and result in serious adverse events, including cardiac adverse event based on former evidences. Thus, CQ or HCQ with or without azithromycin for treating hospitalized (AI) and nonhospitalized (AIII) patients with COVID-19 has not been recommended by the COVID-19 Treatment Guideline Panel (CTGP).

Remarkably, repurposing drugs that might inhibit the entrance of virus into host cell have not shown clinical preference. The main mechanisms of these repurposed drugs remain uncertain, and the interaction sites of new approaches are relatively clear. Next, the structure-based pathogenic mechanisms and new therapeutic strategies of COVID-19 are summarized.

#### Spike glycoprotein

S protein, a highly *N*-glycosylated protein of approximately 180 kDa, has been the most widely studied target in SARS-CoV-2.^64^ The cryo-electron microscopic structure of S protein exists as a homotrimer in the virion envelope, which contains two functional subunits: membrane-distal S1 and membrane-proximal S2 subunits.^11,12^ The former is composed of N-terminal domain (NTD) and RBD, whereas the latter comprises fusion peptide, connector domain (CD), a helix–turn–helix structure formed by heptad repeat 1 (HR1) and heptad repeat 2 (HR2) around a central helix, transmembrane domain (TM), and CT.^65^ The noncovalent bind form of S1 and S2 usually presents in several CoVs before fusion.^66–70^ S1 subunit exerts its effects on recognizing and binding protein-based receptors primarily via RBD.^71^ Thus, the RBD of S protein exerts its effects on binding ACE2 specifically, which is a significant target for antiviral drugs and vaccines.^72–74^ Additionally, NTD is reported to be involved in sugar-based receptor binding, virus attachment, and the S protein transition in pre- or post-fusion.^75^ S2 subunits are responsible for mediating cellular and virus–membrane fusion. Notably, S1 subunit also contributes in stabilizing the prefusion status of biomembrane-anchored S2 subunit.^76^

Owing to the presence of *N*-linked glycan, the S trimer could guarantee proper folding and modulate the interaction of nAbs with host proteases. Therefore, the S protein, particularly the RBD of S protein, has been the potential target for COVID-19 drug development. The majority of these novel drugs have been researched into the clinical trial phase. From the perspective of dispelling SARS-CoV-2, this study focuses on the current licensed vaccines and mAbs for the EUA,^44,77–80^ which have been applied in the clinic, with the hope that these could indicate direction and shed light on ways to tackle SARS-CoV-2.

#### Vaccines of SARS-CoV-2

**Fig. 5:**
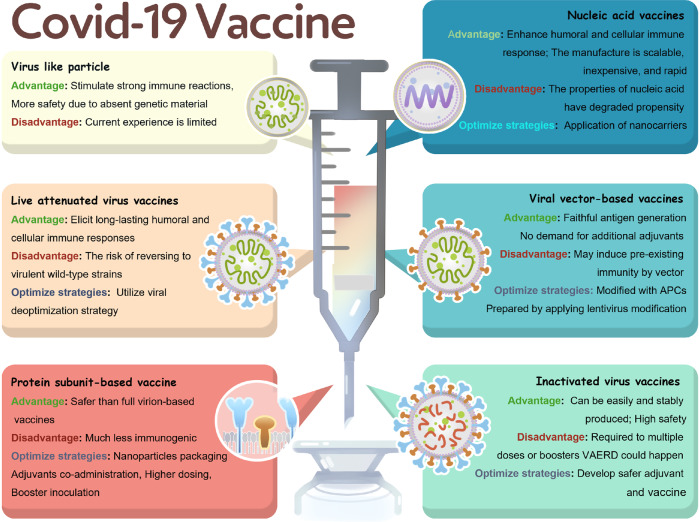
The overview of the vaccine types against COVID-19, including the advantages, disadvantages, and optimal strategies

Since the fast, unprecedented entry of the first SARS-CoV-2 vaccine candidate on March 16, 2020,^81–83^ 216 vaccines underwent preclinical development and 100 are undergoing clinical trial (Supplementary Table 1) worldwide (https://biorender.com/covid-vaccine-tracker) as of May 27, 2021. Presently, more than five kinds of vaccines announced by the Chinese Health Commission are developed for SARS-CoV-2 in China, including influenza viral vector vaccine, adenoviral vector vaccine, inactivated vaccine, nucleic acid vaccine, and subunit protein vaccine. The advantages, disadvantages, and optimal strategies of each type of vaccines have been summarized in Fig. 5. Moreover, 11 vaccines have been licensed or approved for EUA (Table 1). The details of each licensed and EUA vaccines were thoroughly analyzed and compared to provide instructions for the clinical application of these vaccines.

**Table 1:** The characteristics of the licensed and EUA vaccines

Vaccine platform description	License code	Composition	Developers	Country	Licensed date	Approval type	Route of administration	Number of doses	Schedule	Regimen	Efficacy to SARS-CoV-2	Adverse events	Storage condition	Price	Protection duration	Estimated supply
Viral vector-based vaccines	Sputnik V	rAd26-S + rAd5-S	Gamaleya + Russian Federation	Russia	Aug 11, 2020	Licensed	IM	2	Day 0 and Day 21	First dose rAd26, second dose rAd5	91.6%	Flu-like illness (15.2%), injection-site reactions (5.4%), headache, asthenia	Liquid (stored at −18°C) and freeze dried (stored at 2–8 °C)	$10	At least 6 months	500 million
	Covishield	ChAdOx1-S	AstraZeneca + University of Oxford	England	Jan 2021	EUA	IM	2	Day 0 and Day 28	(1) COV001 (UK): a dose of SD (5 × 10^10^ VLPs) with the booster dose; (2) COV002 (UK): LD (2.2 × 10^10^ VLPs) + a SD; (3) COV003 (Brazil): two doses (3.5–6.5 × 10^10^ VLPs); (4) COV004 (South Africa): two doses (3.5–6.5 × 10^10^ VLPs)	All: 66.7%; LD/SD: 80.7%; SD/SD: 63·1%	Severe adverse events (0.2%): infection, hemolytic anemia, transverse myelitis	Stored and distributed at 2–8 °C for 6 months	$4–$8.1	At least 6 months	30 million
	Recombinant novel coronavirus vaccine (Ad5-nCoV)	Ad5-S	CanSino + BIB	China	Feb 25, 2021	Licensed	IM	1	Day 0	5 × 10^10^ VLPs	14 days after injection: 68.83%; 28 days after injection: 65.28%	Injection-site pain (56%), fatigue (42%), fever (32%), headache (29%)	Stored and distributed at 2–8 °C	NA	At least 6 months	5 billion
	Ad26.COV2.S	Ad26-S	Janssen Pharmaceutical	America	Feb 27, 2021	EUA	IM	1	Day 0	5 × 10^10^ VLPs	66%	Injection-site pain (48.6%), headache (38.9%), fatigue, myalgia, nausea	Stored at −20 °C for 2 years, stored and distributed at 2–8 °C for 3 months	$10	At least 6 months	1 billion
RNA-based vaccine	BNT162b2	LNP-formulated, nucleoside-modified RNA	Pfizer/BioNTech	America	Dec 11, 2020	EUA	IM	2	Day 0 and Day 28	30 μg	95%	Injection-site pain (66–78%), fatigue (4%), headache, lymphadenopathy, Bell’s palsy	Shipping (−70 °C); ready to use (2–8 °C) up to 5 days	$19.5	At least 6 months	50 million
	mRNA-1273	LNP-formulated, nucleoside-modified RNA	Moderna	America	Dec 18, 2020	EUA	IM	2	Day 0 and Day 28	100 μg	94.1%	Injection-site pain (60%), fatigue (20%), headache, myalgia, Bell’s palsy	Stored at −20 °C for 6 months, ready to use (2–8 °C) for a month or (room temperature) up to 5 h	$25–$37	At least 6 months	50 million
Inactivated virus	BBIBP-CorV	Inactivated virus (HB02 strain) (Vero cell) with alum as adjuvant	Sinopharm + CNB + BIBP	China	Dec 31, 2020	Licensed	IM	2	Day 0 and Day 21	4 μg/0.5 mL	79.34%	Injection-site pain (35%), fever (6%), swelling, fatigue	Stored and distributed at 2–8°C	Free	At least 6 months	1 billion
	Covaxin	Whole-virion inactivated Vero cell	Bharat Biotech	India	Jan 3, 2021	EUA	IM	2	Day 0 and Day 14	6 μg /0.5 mL	Seroconversion rates: 96.6%	Injection-site pain (3.2%), headache (2%), fatigue, fever	Stored and distributed at 2–8 °C	NA	At least 6 months	300 million
	CoronaVac	Inactivated virus (CN02 strain) (Vero cell) with alum as adjuvant	Sinovac	China	Feb 5, 2021	Licensed	IM	2	day 0 and day 14	3 μg/0.5 mL	Brazil: 50.7%; Chile: 67%; Turkish: 91.25%	Injection-site pain (17%), diarrhea (5%), fatigue, fever, headache	Stored and distributed at 2–8 °C for 6 months	Free	at least 6 months	2 billion
Protein subunit	WIBP-CorV	Inactivated virus (WIV04 strain) (Vero cell) with alum as adjuvant	Sinopharm + CNB + WIBP	China	Feb 25, 2021	Licensed	IM	2	day 0 and day 21	5 μg/0.5 mL	72.51%	Injection-site pain (14.3%), fever (2.4%), diarrhea, fatigue, swelling	Stored and distributed at 2–8 °C	NA	at least 6 months	1 billion
	ZF2001	An RBD-dimer protein produced in Chinese hamster ovary (CHO) cells with alum as adjuvant	Anhui Zhifei Longcom Biopharmaceutical + Institute of Microbiology, Chinese Academy of Sciences	China	Mar 10, 2021	EUA	IM	3	Day 0 + 28 + 56	25 μg/0.5 mL	Seroconversion rates: 97%	Itch (19%), redness (16%), swelling, injection-site pain, fever	Stored and distributed at 2–8 °C	NA	at least 6 months	300 million

*rAd5* recombinant adenovirus 5, *rAd26* recombinant adenovirus 26, *S* Spike (protein), *ChAdOx1-S* attenuated resulting in infections in chimpanzees, *LNP* lipid-based nanoparticles, *BIB* Beijing Institute of Biotechnology, *BIBP* Beijing Institute of Biological Product, *WIBP* Wuhan Institute of Biological Product, *CNB* China National Biotec Group, *EUA* emergency use authorization; *LD* low dose; *SD* standard dose, *BD* booster dose, *VLP* virus-like particles

To date, 11 vaccines for SARS-CoV-2 have been licensed or approved by EUA worldwide; these vaccines are of the following four types: viral vector-based vaccine, RNA-based vaccine, inactivated virus vaccine, and protein subunit vaccine. Virus-like particle vaccines may still need time to evaluate their efficacy and safety owing to the temporarily insufficient progress of clinical trials. The licensed vaccines are Sputnik V in Russia and Ad5-nCoV and three inactivated vaccines in China. The EUA vaccines include BNT162b2, mRNA-1273, and Ad26.COV2.S in America; Covishield in England; Covaxin in India; and ZF2001 in China.

##### Efficacy and safety

The efficacy and safety of developing vaccines against COVID-19 should be given an overarching priority. Among these 11 vaccines, BNT162b2 was developed by Pfizer/BioNTech^84^ and mRNA-1273^85^ was developed by Moderna, with the highest efficacy at 95% and the second highest efficacy at 94.1%, respectively; these are RNA-based vaccines. The most common adverse event of these two RNA-based vaccines was injection-site pain, which was slightly higher in BNT162b2 (66–78%) than in mRNA-1273 (60%). As the first licensed vaccine, on the basis of Ad26 and Ad5, Sputnik V^86^ displayed the third highest efficacy at 91.6% with the largest adverse response proportion of flu-like illness presented at 15.2%. Although the production of Sputnik V was criticized for absence of transparency, corner cutting, and unseemly haste^87,88^ at first, the positive results of phase III clinical trials demonstrated the scientific and clear principle vaccination, which suggests the potential of reducing the incidence of SARS-CoV-2.

In terms of inactivated virus vaccines, three of the four licensed vaccines are from China. The main differences among these three vaccines are the different virus strains derived from different patients. CoronaVac developed by Sinovac uses CN02 strain, whereas SARS-CoV-2 vaccines (Vero Cell) developed by the Beijing Institute of Biological Product (BIBP) and Wuhan Institute of Biological Product (WIBP) used HB02 strain and WIV04 strain, respectively.^89,90^ The highest overall protective efficacy shown by SARS-CoV-2 vaccines (Vero Cell) was developed by BIBP (BBIBP-CorV) at 79.34%. Sinovac conducted the phase III clinical protocol in Brazil, Chile, Indonesia, and Turkey. The results in Turkey showed more favorable efficacy at 91.25% than those in Chile at 67% and Brazil at 50.65%.^91^ Although the same batch and immunization schedule of vaccines were applied in these four countries, the significant difference, which was evident in the efficacy, may be owing to the distinct race characteristics. SARS-CoV-2 vaccine (Vero Cell) produced by WIBP was licensed recently on February 25, 2021 for which the efficacy obtained was 72.51%. Among these three vaccines developed in China, WIBP-CorV displayed the smallest proportion of the most common and the second common adverse reactions, comprising injection-site pain and fever at 14.3 and 2.4%, respectively. Apart from these, diarrhea, fatigue, swelling, and headache have been reported with low incidence among the adverse events of these three vaccines. Covaxin (BBV152) is engineered by Bharat Biotech in India (https://www.astrazeneca.com/covid-19.html); however, the accurate results of the phase III clinical trial or the efficacy of the vaccine have not been disclosed yet. The published results of phase II clinical trial showed 96.6% seroconversion rate and a significantly lower incidence of adverse events than the other anti-SARS-CoV-2 vaccines.^92^ Clinical I trial of Covaxin showed that only 15% of recipients suffered from side effects with injection-site pain (3.2%), followed by headache, fatigue, and fever. Longer follow-up should be considered to evaluate the safety and efficacy of Covaxin.

Regarding viral vector-based vaccines, Covishield (AZD1222), developed by AstraZeneca and the University of Oxford, was approved by EUA in England (https://www.astrazeneca.com/covid-19.html). The phase III clinical trial of Covishield in the UK, Brazil, and South Africa^93,94^ showed the overall efficacy obtained at 66.7%. The efficacy in patients who received a low dose (LD) (2.2 × 10^10^ virus-like particles (VLPs)) followed by a standard dose (SD) (5 × 10^10^ VLPs) was 80.7%, whereas that in patients injected with two SDs was 63.1%. Furthermore, the trial recommended 3 months to be the injection interval between two doses, which achieved superior vaccine efficacy of 81.3% than that of ≤6 weeks. This means that participants who received immunization schedule of LD/SD Covishield with an interval of 3 months would harvest a favorable protective efficacy. Moreover, the incidence of severe adverse events was reported to be <0.2%, among which infection was the most common.

Recently, a recombinant tandem-repeat dimeric RBD protein vaccine (ZF2001), produced in CHO cells by the Anhui Zhifei Longcom Biopharmaceutical Company,^95^ was approved for EUA worldwide. The completed phase II trial^96^ in adults aged 18–59 years revealed that this vaccine was well tolerated without severe adverse responses and could stimulate moderate cell immune responses, owing to the balanced generation of TH1/TH2 cell-related cytokines. The seroconversion rate was achieved at 97% with the administration of 25 μg 2 weeks after the third dose. Additionally, itch (19%) and redness (16%) were the most frequent adverse events during the injection. Furthermore, the clinical trials that recruited older participants (NCT0455035) and individuals belonging to multiple ethnic backgrounds (NCT04646590) cohort are ongoing.

The abovementioned licensed or EUA vaccines are two- or three-dose vaccines, with the injection interval varying from 14 to 21 days. However, one-dose vaccines have been designed with more convenience for the public. Experts claimed that the single-dose vaccine could provide efficacy equal to that of two-dose^97^ vaccine, which suggested that a single-dose could cover twice as many people as a double dose with the same protection and capacity.

The recombinant novel coronavirus vaccine (Ad5-nCoV) developed by CanSino is a single-dose vaccine. According to the interim analysis of clinical III trial, one-dose Ad5-nCoV showed 68.83 and 65.28% efficacy 14 and 28 days after injection, respectively (https://www.astrazeneca.com/covid-19.html). Injection-site pain (56%), fatigue (42%), fever (32%), and headache (29%) were the common reported adverse reactions.^98^

Subsequently, Ad26.COV2.S developed by Janssen Pharmaceutical got the EUA in America. This single-shot vaccine has shown 72% efficacy in the US and 66% overall efficacy at preventing moderate-to-severe COVID-19 after 28 days of injection.^99^ Regarding the safety data of Ad26.COV2.S, overall fever rates were reported at 9% and grade 3 fever was accounted at 0.2%.

##### Storage condition, price, protection duration, and estimated supply

RNA-based vaccines have more strict storage condition than other vaccines, which suggests the greater difficulty in transporting and large-scale promoting. BNT162b2 has the most strict storage temperature of −70 °C. mRNA-1273 could be stored at −20 °C for 6 months in an ordinary refrigerator maintained at 2–8 °C for a month and even at room temperature for up to 5 h. The other vaccines could universally be stored at 2–8 °C for 6 months, thereby greatly enhancing their universality.

The price of the vaccines disclosed on the Internet may not be the final price when released. Considering the present data, Covishield presented the lowest price at $4–$8.1, and it has been granted conditional marketing authorization or emergency use in >50 countries. At present, World Health Organization (WHO) will accelerate the access to the vaccine in up to 142 countries through COVID-19 Vaccine Global Access (Covax) (https://www.astrazeneca.com/covid-19.html).

According to the WHO target product profiles for SARS-CoV-2 vaccines,^100^ the protection duration is required for at least 6 months. Currently, no exact duration data of the licensed or EUA vaccines has been published online, and further evaluation remains to be performed.

All the vaccine companies begin to ramp up the production after the approval. CanSino proposed to supply 5 million vaccines during 2021, the highest production of the estimated supply. Janssen, Sinopharm/BIBP, Sinopharm/WIBP, and Sinovac stated that 1 million supply could be utilized in this year.

#### mAbs of SARS-CoV-2

##### Bamlanivimab

Bamlanivimab, also known as LY3819253 and LY-CoV555, is a neutralizing mAb that binds to the RBD of the S protein of SARS-CoV-2.^101–103^ A randomized controlled phase I/II trial (BLAZE-1 study) compared bamlanivimab (three doses: 700, 2800, and 7000 mg) with placebo.^103^ The primary outcome was SARS-CoV-2 virus load reduction from day 1 to day 11. The results showed that antibody induced by 2800-mg dose experienced significant decrease than that induced by placebo. Meanwhile, the 700- and 7000-mg groups had no tendency of notable reduction, possibly because these patients had been effectively cleared from SARS-CoV-2 before day 11. The most common adverse event of bamlanivimab was nausea (3.9%), followed by dizziness (3.2%) and moderate infusion responses (2.3%). Bamlanivimab group showed decreased severity of symptoms and hospitalization proportion compared with the placebo group. On November 10, 2020, bamlanivimab was issued EUA for patients with mild-to-moderate COVID-19 (pediatric and adults).^104^ The authorized administration is the single 700-mg dose with vein injection infusion for >60 min. If a patient tests positive for SARS-CoV-2 or the onset of symptoms of infection was <10 days, this drug should be utilized as soon as possible^105^ (BIIa).

##### Bamlanivimab plus etesevimab

Bamlanivimab and etesevimab (LY-CoV016) are neutralizing mAbs that target different but overlapping epitopes in the RBD of the S protein of SARS-CoV-2.^106^ A randomized controlled phase III trial (BLAZE-1 study) included >1000 participants and compared bamlanivimab plus etesevimab with placebo.^107,108^ The results suggested that the participants who received bamlanivimab plus etesevimab had a 70% relative reduction and a 5% absolute reduction in Covid-19-related hospitalizations or death from any cause compared with those in the placebo group (*p* < 0.001). Endpoint events (hospitalization or death by day 29) occurred in 2% of the participants in the bamlanivimab plus etesevimab group and 7% in the placebo group. The BLAZE-4 trial focused on the dose of bamlanivimab and etesevimab.^107^ Furthermore, the FDA selected bamlanivimab 700 mg and etesevimab 1400 mg to be the authorized dose for patients with mild-to-moderate COVID-19.^109^ This dosage was subsequently studied in a new BLAZE-1 trial. The bamlanivimab and etesevimab group also showed superior death and hospitalization rate than the placebo group. On March 5, 2021, the European Medicines Agency has allowed EU Member States to utilize bamlanivimab plus etesevimab for emergency use in patients with COVID-19.

##### Casirivimab plus imdevimab

Casirivimab (REGN10933) and imdevimab (REGN10987) constitute a combined cocktail (REGN-COV2) that targets the RBD of the S protein of SARS-CoV-2.^80^ A randomized controlled phase I/II trial (R10933-10987-COV-2067 study) compared REGN-COV2 antibody with placebo.^110^ An interim analysis of this study indicated that the combination of casirivimab and imdevimab may have a greater effect in patients who test negative for SARS-CoV-2 serum antibodies at baseline. The proportion of patients who had at least one COVID-19-related medical visit was lower in the casirivimab plus imdevimab group (3%) than in the placebo group (6%).^110^ Based on the results, the FDA issued EUAs to use casirivimab plus imdevimab in outpatients with mild-to-moderate COVID-19^111^ (BIIa). The authorized dosage for both casirivimab and imdevimab were 1200 mg intravenous (IV) infusion for over 1 h. Present studies have no evidence of the comparison of the casirivimab and imdevimab with bamlanivimab and etesevimab. More details concerning the comparison remain to be determined.

#### S protein in SARS-CoV variants

D614G mutation of S protein was found with increased transmissibility, which played a predominant role early in the COVID-19 pandemic.^112,113^ However, among vaccinated individuals and patients with COVID-19, this mutation showed a mild effect on neutralizing their sera.^114^ Recently, several variants of SARS-CoV-2 with increased transmissibility have emerged worldwide, compromising virus control and raising concerns that the unknown and constant mutations might weaken current efforts on combating the pandemic. Therefore, three main SARS-CoV-2 variants that caused the outbreak have been summarized in this study, and whether current available therapy could fight against viral infection sequentially has been illustrated. Moreover, other potential therapies preventing reinfection by new variants are summarized as follows:

The variant B.1.1.7 of SARS-CoV-2 (UK variant), also named as 501Y.V1 or variant of concern 202012/01, first emerged in England, has caused a surge in COVID-19 cases.^115^ This variant has been reported to be spread to >50 countries and seems to become virulent in the future.^116–118^ It has eight S protein mutations except for D614G.^119^ SARS-CoV-2 B.1.351 (501Y.V2) and P.1 (501Y.V3), also termed as South Africa variant and Brazil variant, respectively, were claimed to have more strong infectious ability. These three variants share the N501Y mutation in RBD, which is associated with enhanced transmissibility. B.1.351 and P.1 variants, respectively, harbor 9 and 11 exchanges, including N501Y, E484K, and K417N (B.1.351)/T (P.1) mutations in the RBD. Additionally, B.1.1.7 has 69–70 and 144 deletions and B.1.351 has 242–244 deletions in NTD, both of which could damage the antibodies’ binding sites in NTD.^120,121^ Although P.1 variant lacks NTD deletions,^122^ it could also be studded with point mutations in this area, which might harbor similar functional performances. Because majority of mutations are located in the ACE2-binding site (RBD) or the antigenic supersite in NTD,^120,121^ which are the potential targets of virus nAbs, the efficacy of vaccines and mAb therapies could be impaired by these variants.^119^ In fact, the susceptibility to therapy-mediated reaction varied between SARS-CoV-2 wild type (WT) and the other three variants. However, previous evidence demonstrated that no major differences were found in the entry kinetics of the virus, efficiency of virus–cell and cell–cell fusion, and stability of the S protein between SARS-CoV-2 WT and variants B.1.1.7, B.1.351, and P.1.^123^

#### Vaccine sera

As the extensively utilized therapy, vaccines are administered with great expectations in combating with SARS-CoV-2 variants. Indeed, vaccine antigens utilizing the full-length S protein, containing S-mRNA and S-subunit vaccines, have shown different neutralization activity toward the three variants.^124,125^

Regarding mRNA vaccines, several studies^123,126–128^ reported that serum from individuals vaccinated with BNT162b2 and mRNA-1273 could efficiently neutralize B.1.1.7 spike protein (SP) in pseudoparticles.^129,130^ Although B.1.1.7 strains presented with additional mutations (N501Y + 69/70-deletion), they could be neutralized robustly by BNT162b2-induced antibodies.^131^ However, B.1.351 and P.1 variants’ neutralization was found to be reduced^128^ significantly in BNT162b2 and mRNA-1273 vaccines. Currently, mRNA-1273.351 has been studied against B.1.351 in phase I clinical trial (NCT04785144). Similar results were presented with Sputnik V Ad26/Ad5 vaccine.^132^ The sera from inoculated participants demonstrated the efficacy of neutralizing B.1.1.7S protein and mildly decreased activity in combating only E484K-substituted S protein.^133^ Inversely, B.1.351 failed to be neutralized by Sputnik V Ad26/Ad5 vaccine. Additionally, both the AZD1222 and NVX-CoV2373 vaccines could provide protection for B.1.1.7 variant.^134,135^ Janssen, Novavax, and AZD1222 vaccines showed a marked reduction in efficacy for B.1.351 variant, whereas the first two still presented over 50% protective efficacy for moderate and severe disease.^136^ However, efficacy of AZD1222 was approximately 10% in fighting with B.1.351-caused mild-to-moderate disease, and no efficacy was demonstrated against severe disease in a phase II trial.^137,138^ The neutralizing geometric mean titers (GMTs) against P.1 variant for AZD1222 showed similarity with those against B.1.1.7 variant and considerable superiority to those against B.1.351 variant.^136^ Apart from these, Ad26.COV2.S vaccine regimen, which was applied for aged nonhuman primates, showed maintained neutralization for B.1.1.7 lineage and reduced neutralization for B.1.351 lineages.^139^

Vaccines can be more beneficial when they utilize immunogens, which produce and enrich RBD-targeted nAbs. It shows more resistance to the variants of SARS-CoV-2 with their multiple RBD-binding models, thus protecting broader spectrum of virus variant. Naturally, RBD-based vaccines increase concerns for researchers.

ZF2001, as an RBD-recombinant vaccine, has been studied for its effectiveness against SARS-CoV-2 variants. Huang et al.^140^ evaluated the neutralization activity in ZF2001-induced (*n* = 12) and BBIBP-CorV (*n* = 12) serum nAbs against SARS-CoV-2 B.1.351. They found that the variant B.1.351 could not escape the immunity induced by these two vaccines. However, when the GMTs are reduced 1.5–1.6 times, the clinical efficacy of ZF2001 and BBIBP-CorV could also be influenced. Another study conducted by Cao et al.^122^ revealed that ZF2001 vaccines had double tolerant ability for combating SARS-CoV-2 B.1.351 than CoronaVac vaccines in authentic or pseudovirus assays. Notably, half-maximal neutralizing titer (NT50) reduction was found less in the extended three-dose (0/30/140 days) than in the standard three-dose (0/30/60 days) ZF2001 group, which may be attributed to the extra antibody maturity induced by constant hypermutations before the boost of the third dose.^141^ ZF2001 with an extended three dose could motivate enhanced neutralization activity so that it could counter 501Y.V2 utilizing a suitable third-dose boost.

In fact, because various experimental designs of neutralization assays are performed using pseudovirus, comparing the neutralization fold changes among different types of vaccines is difficult. However, the efficacy trend is similar, i.e., B.1.1.7 variant has the least possibility to escape from the neutralization antibodies induced by the licensed or EUA vaccines, followed by P.1 and B.1.351 variants. With the additive effect of E484K and 242–244Δ, B.1.351 presented with the most significant reduction of neutralization reaction. Moreover, several studies suggested that B.1.351 with full suite of mutations could decrease the immunological surveillance substantially including only three RBD exchanges (N501Y, E484K, and K417N) owing to the non-RBD changes.^142^ Therefore, developing vaccines against B.1.351 should be given the highest priority. Considering that P.1 showed similar RBD exchanges with B.1.351 but with less impaired neutralization, implying no widespread escape presentation, the ancestral/parent strains may protect from P.1 continuously. Currently, RBD-based vaccines are considered ideal for countering potential NTD mutations, especially the vaccines with the third booster shot.^122^ The combination of the variant vaccines and the current vaccines (bivalent vaccines) could also be considered. Before the violent spread of the variants, rapid deployment of WT antigen vaccines may help in putting an end to the pandemic.

#### Monoclonal antibodies

Several researches have illustrated the resistant effect of mAbs on B.1.1.7, B.1.351, and P.1 variants.^143–145^ B.1.1.7 variant is refractory to the neutralization by NTD supersite-directed mAbs,^119^ which is largely conferred by 144 deletion. B.1.351 resistance largely depends on the R246I and/or 242–244 deletions. All 144 and 242–244 deletions and R246I fall within the supersite of NTD.^120,121^ P.1 does not have NTD deletions but NTD mutations (R190S, D138Y, P26S, T20N, and L18F), which could influence the binding of mAbs. Notably, these EUA mAbs targeting RBD are majorly involved in B.1.351 and P.1 resistance.

Casirivimab (REGN10933) could partially inhibit virus entry of B.1.351 and P.1 variants, in line with the mutations in the antibody-binding site of the S protein. Moreover, the neutralization ability of casirivimab could be severely damaged (773-fold), whereas that of imdevimab was unaffected by B.1.351.^124^ The EUA antibody cocktail (REGN-COV2), combining casirivimab with imdevimab (REGN10987), could restore efficient suppression, manifesting the suitability of this regimen for B.1.351 and P.1 infection. Conversely, another EUA antibody for SARS-CoV-2, bamlanivimab, failed to inhibit entry driven by B.1.351 and P.1 S protein, which is according to the E484K mutation in the antibody-binding region.^119^

To date, the utilization of the current mAbs, including casirivimab and/or imdevimab, may provide partial protection for the SARS-CoV-2 variants. However, owing to the absence of the large-scale clinical evidence, the efficacy of mAbs against variants still needs to be explored. Meanwhile, virus genomic surveillance worldwide and next-generation antibody treatment promotion should highlight their importance, including the combination that targets distinct antigen epitopes.

### Antireplication

The other kind of antiviral drugs targets viral replication. Nsps mainly function during this progress. Approximately 67% of the SARS-CoV-2 genome comprises 5’-ORFs-1a/1b (ORF) that encodes two polyproteins: polyprotein 1a (pp1a) and polyprotein 1 ab (pp1ab). These proteins are degraded into 16 Nsps, also called Nsp 1–16.^20^ Nsps, including RdRp (Nsp12), 3-chymotrypsin-like protease or main protease (3CL_pro_ or M_pro,_ Nsp5), and papain-like protease (PL_pro_, Nsp3), play a vital function in the life cycle of SARS-Cov-2, particularly in replication. Furthermore, other Nsps participate in the process of viral replication. In this part, the characteristics of these Nsps and their potential in COVID-19 treatment will be discussed in detail.

#### The RNA-dependent RNA polymerase

**Fig. 6:**
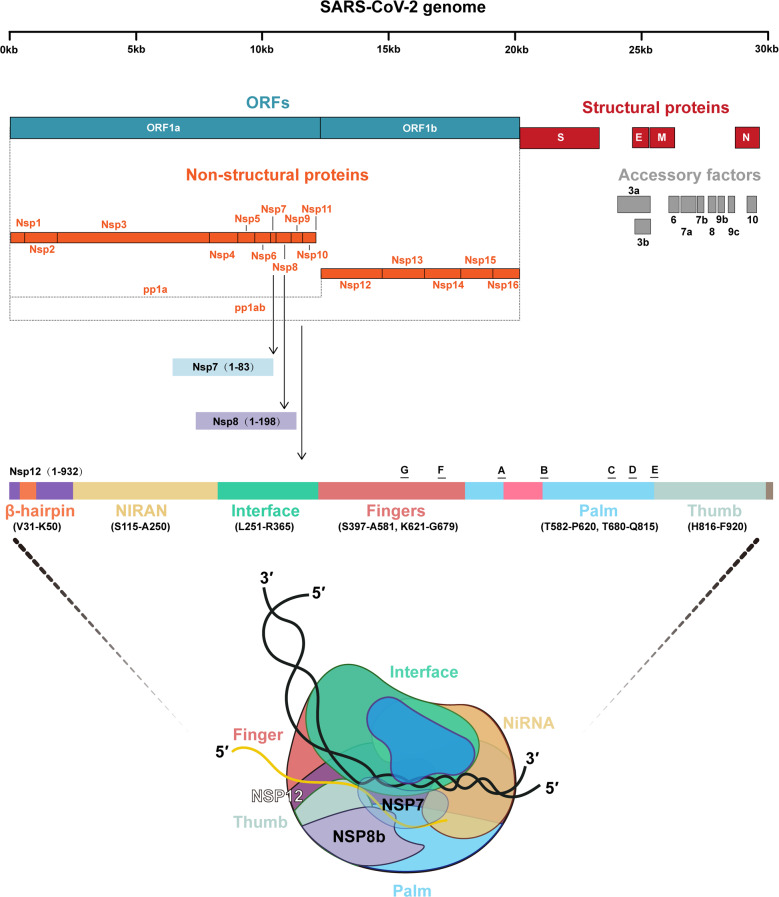
The genome of SARS-CoV-2 comprises approximately 29,900 base pairs, containing a 5’ cap structure and a 3’ ploy (A) tail, with 11 ORFs. ORF1ab occupies approximately two-third of the genome encoding 16 nonstructural proteins (Nsps). The RNA polymerase complex comprises Nsp12 (RdRp), Nsp7, and Nsp8. The RdRp domain is the core of the RTC that is composed of three subdomains named finger, palm, and thumb

Because RdRp (Nsp12) is a protein specifically present in the virus and without host cell homologs, it is considered to be superior target for developing a safer and more efficient treatment approach.^146^ RNA viruses encode RdRp for the transcription and replication of viral genome. Meanwhile, RdRp alone has low efficacy in combining with template-primer RNA. The replication/transcription complex (RTC) of SARS-CoV-2 contains not only RdRp but also other two subunits, Nsp7 and Nsp8.^147^ The RdRp domain is the core of the RTC that comprises three subdomains, namely, finger, palm, and thumb. Nsp7 binds to the thumb subdomain, and Nsp8 binds to the thumb subdomain and finger domain.^148^ Nsp7 and Nsp8 significantly improve the binding of Nsp12 and RNA (Fig. 6). Some key amino acid residues of SARS-CoV-2 RdRp have structure similar to those of several other positive-sense RNA viruses, including hepatitis C virus (HCV), Zika virus, and coronavirus (SARS, MERS),^149,150^ whereas several key points can distinguish them, including the nidovirus RdRp-associated nucleotidyltransferase. However, there is not sufficient evidence to evaluate how this difference affects the effectiveness of nucleotide analog medicines for COVID-19.

Several drugs that inhibit RdRp and have been approved in other infected diseases were considered to be repurposed in COVID-19. Remdesivir, once approved to be used for Ebola virus treatment,^56,151^ is proved to be effective in COVID-19 by targeting Nsp12 and inhibiting the synthesis of viral RNA.^152^ It has a 1’-cyano-substituted adenosine nucleotide that mimics and transfers into active RDV-TP in the body.^153^ RDV-TP is proposed to inhibit the viral RdRp through nonobligate RNA chain termination. Several large-scale clinical trials have evaluated the safety and efficacy of remdesivir in treating COVID-19.^154^ In ACTT-1 trial, remdesivir reduced the time to clinical recovery in patients with severe disease^154^ and has improved outcomes in hospitalized patients with moderate COVID-19 compared with standard of care. FDA has approved the use of remdesivir to treat COVID-19 in hospitalized patients (age ≥12 years and weight ≥40 kg). According to the CTGP, remdesivir is recommended in hospitalized patients who need supplemental oxygen (BIIa). The main side effects of remdesivir include elevated transaminase levels, gastrointestinal symptoms (e.g., nausea), increased prothrombin time, and hypersensitivity reactions.

Similar to remdesivir, nucleotide analog drugs, including ribavirin and favipiravir, inhibit the transcription of viral RNA by mimicking RNA nucleotide and covalently linking to the replicating RNA. Ribavirin, a guanine analog, is also a type of RdRp inhibitor. It is widely used in hepatitis C and human respiratory fusion virus infection. Its antivirus activity to other coronaviruses makes it a drug candidate for COVID-19 treatment.^154^ On the clinicaltrial.gov website, two trials on ribavirin are recruiting, among which one has been completed. The published data have shown that early triple combination of ribavirin, IFNβ-1b, and lopinavir/ritonavir was safe and effective in patients with mild-to-moderate COVID-19 compared with lopinavir/ritonavir alone with respect to controlling symptoms, promoting viral shedding, and shortening hospital stay.^155^ However, it was a small-scale clinical trial with 127 patients and was not enough to confirm the effect of ribavirin on SARS-CoV-2. Ribavirin causes severe dose-dependent hematological toxicity. Red blood cells in the human body lack dephosphorylated enzymes. The phosphorylated ribavirin accumulates in red blood cells, resulting in a high concentration, which ultimately changes the fluidity of red blood cell membranes, leading to hemolytic anemia.^156,157^ It also has strong reproductive toxicity that can cause fetal anomalies. Thus, ribavirin was not recommended in COVID-19 treatment.^158,159^ Other RdRp inhibitors are under research, but no positive results have been gained to date. Favipiravir^160^ has been approved for the treatment of influenza virus and showed a promise in Ebola virus treatment.^161–163^ On the clinicaltrial.gov. website, 31 clinical trials of favipiravir for COVID-19 treatment are active. The published data were small scale, and the results of the effectiveness of favipiravir in COVID-19 were controversial and still need to be confirmed in further clinical trials.^164,165^

Because Nsp12–Nsp7–Nsp8 complex works to prolong viral RNA, compounds that interrupt their binding are potential drugs against COVID-19. After docking and virtual screening of RTC structures, a total of eight compounds (i.e., nilotinib, saquinavir, lonafarnib, tegobuvir, cepharanthine, filibuvir, tipranavir, and olysio) were selected as candidates to battle SARS-CoV-2, but no further preclinical or clinical studies were conducted.

#### The 3-chymotrypsin-like protease or main protease

3-chymotrypsin-like protease or main protease (M_pro_, Nsp5) is involved in the replication and transcription of viral genes. M_pro_, similar to a knife, cuts the viral-translated polyproteins into functional proteins. M_pro_ possesses >11 action sites on the pp1ab, and their most recognition sequence is Leu-Gln ↓ (Ser, Ala, Gly) (↓ marks the cleavage site). Replication would stop without M_pro_. Considering the essential functions in the virus and lack of homologous series in host cells, M_pro_ is believed to be a candidate target to fight against SARS-CoV-2.^166,167^ However, there is no protease inhibitor of M_pro_ with satisfactory effect to date. Lopinavir/ritonavir, approved to be used in HIV, was thought to inhibit the M_pro_ but has shown no benefit in clinical practice.^168^ Lopinavir and ritonavir are antiretroviral protease inhibitors, which were approved as combination therapy in the treatment of HIV infection. Lopinavir functions as a specific inhibitor of HIV-1 protease that prevents HIV-1 replication in host cells and blocks the infection of HIV-1. The combination of ritonavir decreases the hepatic metabolism of lopinavir and enhances its efficacy. Lopinavir showed inhibition of coronavirus (MERS-CoV and SARS-CoV) replication in in vitro experiments.^169,170^ In the clinicaltrials.gov website, 22 interventional clinical trials of lopinavir/ritonavir in COVID-19 are ongoing or completed. However, to date, no clinical results have been presented to support the use of lopinavir/ritonavir or other HIV protease inhibitors in COVID-19. Both the large-scale multicenter clinical trials RECOVERY and Solidarity Trial suggested no preference of lopinavir/ritonavir compared with standard care.^168,171^ The unsatisfactory results of lopinavir/ritonavir against SARS-CoV-2 can be because the protease of SARS-CoV-2 is different from that of retrovirus (the aspartic and chymotrypsin-like protease families, respectively).^172^ Additionally, the plasma drug concentration achieved with the typical dose of lopinavir/ritonavir is far below the level required to inhibit SARS-CoV-2 replication.^173^ Other antiretroviral drugs were identified to be effective through enzyme activity screening^174^ but failed in clinical practice, including darunavir/cobicistat. Based on the abovementioned evidences, CTGP recommends against the use of HIV protease inhibitors, including lopinavir/ritonavir, for the treatment of COVID-19 in hospitalized patients (AI) and nonhospitalized patients (AIII).

Despite the failure of protease inhibitors in clinical trials, multiple preclinical researches have continued putting in efforts. At the beginning, structure-based virtual and high-throughput screening was used for drug selection. High-throughput drug screening and in vitro study showed that boceprevir, approved for treating anti-HCV, and GC376, a preclinical inhibitor designed to treat feline infectious peritonitis (corona) virus, can suppress M_pro_ activity and SARS-CoV-2 in vitro. Zhang et al.^175^ synthesized peptidomimetic α-ketoamides, a broad-spectrum inhibitor of the Μ_pro_ of β-CoV, α-CoV, and enteroviruses. The concentration for 50% of the maximal effect (EC50) for MERS-CoV in Huh7 cells was 400 pM, and it also had low μM EC50 values for SARS-CoV and enterovirus. Recently, they declared the M_pro_ X-ray structures. With α-ketoamide as reference, adding the P3–P2 amide into a pyridone ring to enhance the half-period of the compound in serum is also an alternative to improve drug efficacy. Recently, in the BSL-2 laboratory, the cell-based luciferase complementation reporter assay has been established to select SARS-CoV-2 M_pro_ inhibitors.^176^ It can easily distinguish actual M_pro_ inhibition from cytotoxicity, thereby significantly improving screening efficacy. Five inhibitors, including Z-FA-FMK, boceprevir, calpain inhibitor XII, GRL-0496, and GC376, have been identified through this method. However, these drugs have not been clinically tested.

### Antiviral release

The process of viral release usually occurs through three ways: host cell lysis, budding, or exocytosis. Oseltamivir is a prodrug against neuraminidase inhibitor, approved for the treatment and prophylaxis of influenza A and B.^177^ Mechanistically, the lipophilic side chain of oseltamivir metabolites binds to the hydrophobic pocket of the active site of the viral neuraminidase to impair the ability of neuraminidase to cleave sialic acid residues on the surface of the infected host cells. It inhibits the release of progeny virion by budding from the infected cells.^178^ Eight clinical trials on oseltamivir and COVID-19 are registered in clinicaltrial.gov., and none of them has been marked as complete. Therefore, the data of oseltamivir in COVID-19 are insufficient.

## Target host cell

As host factors are important regulators of SARS-CoV-2 infection, they are potential targets for antiviral therapy. Hence, the discovery of novel host genes or proteins and related signaling pathways that mediate pathogenesis of COVID-19 is a critical resource that may help us understand the exact biological pathogenesis of this disease based on host factors and may reveal host-directed therapeutic targets against SARS-CoV-2 infection.

### Receptors in host cells impact the viral entry

#### Angiotensin-converting enzyme-2

The ACE2 gene precisely maps to chromosome Xp22 comprising 20 introns and 18 exons, spans 39.98 kb of genomic DNA, generating 6 variants via alternative splicing,^179^ and encodes a type I membrane-bound glycoprotein, ACE2. ACE2 is a homolog of ACE. It comprises 805 amino acids and includes a C-terminal transmembrane anchoring region (carboxy-terminal domain), N-terminal signal peptide region, and a conserved HEXXH zinc-binding metalloprotease motif (catalytic domain).^180^ Although SARS-CoV mainly infects macrophages, pneumocytes, and the lungs,^181^ ACE2 expression is not limited to the lungs and involves the extrapulmonary tissues.^182–184^ Analysis of the expression level of ACE2 in animal models and the evaluation of the human transcriptome using data from different databases indicated that it is high in the small intestine, kidney, colon, testis, thyroid gland, and heart muscle,^185,186^ whereas it is extremely low in the lung, with no expression in the blood cells.^187,188^ This explains why people affected by COVID-19 suffer from gastrointestinal dysfunction and kidney problems.^189,190^ It has a wide range of biological activities, and the main function is to regulate the renin–angiotensin system (RAS) in several diseases.^180,191,192^ Regarding infection with coronaviruses, the virus makes use of the host receptors as a doorway for entry into the host cell. The S proteins of SARS-CoV-2 binding to the human ACE2 for entry into the host cell make the ACE2 a druggable target for COVID-19.^193^

Being a host receptor, ACE2 is commonly localized on the plasma membrane (mACE2). Its N-terminal comprises the catalytic site protruding from the extracellular environment, with multiple active peptides present in the interstitium as substrates. ACE2 can be hydrolyzed by diverse proteases, including TMPRSS2, a disintegrin and metalloproteinase domain-containing protein 10 (ADAM10), and ADAM17. The S1 subunit of the SARS-CoV-2 S protein binds to the ACE2 receptor and then triggers the cleavage of ACE2 by tumor necrosis factor-alpha-converting enzyme (TACE)/ADAM17 at the ectodomain sites,^194^ producing a soluble form to maintain its catalytic activity (sACE2).^195^ Notably, in both in vitro and in vivo experiments, TACE inhibitors can reduce viral entry, demonstrating their essential role in determining SARS-CoV infectivity and their potential use as targets for antiviral treatments. Meanwhile, ACE2 can be shed from the cell and then released into the circulation by ADAM17 while maintaining its catalytic activity and its ability to bind with SARS-CoV-2. In the context of the COVID-19 pandemic, understanding the mechanisms of ACE2 shedding, sACE2 function, and sACE2 plasma level can contribute to the improvement in therapy and diagnosis to track infection progression. The researchers suggested the use of human recombinant ACE2 (hrACE2) protein to saturate the viral S protein and then restrain SARS-Cov-2 cellular entry.^196^ Additionally, the soluble hrACE2 (shrACE2) has attractive physiological characteristics because it can inactivate SARS-CoV-2 present in the extracellular environment. Unlike anti-inflammatory or antiviral therapies, shrACE2 can decrease the binding between mACE2 and SARS-CoV-2 and reduce infectivity.^197^ Additionally, shrACE2 can offset the elevation of LDEABK/DEABK and Ang-II preserving lung function. Administration of hrACE2 is well tolerated in healthy subjects,^198^ and it has been successfully available in treating patients with ARDS.^199^ Moreover, shrACE2 can reduce the infection with SARS-CoV-2 in vitro^200^ and the delivery of shrACE2 could decrease protease degradation^201^ as has already been demonstrated. APN01 is a fully glycosylated rhACE2 and presents a stable noncovalent homodimer.^199^ Although our understanding of the role of endogenous sACE2 in human physiology remains limited, the abovementioned studies have demonstrated that shrACE2 could be an effective drug for the treatment of SARS-CoV-2 infection. Other potential therapeutic strategies, which are targeting ACE2, include blocking the surface ACE2 receptor using anti-ACE2 peptides or antibody.^198^ In a recent research, authors used a single-chain antibody fragment (scFv) or antibody to bind ACE2 and block the interaction between the S protein and ACE2.^202^ Additionally, as mentioned above, the main function is to regulate the RAS in several diseases.^180,191,192^ After viral infection, ACE2 downregulation in organs can disturb the balance between the RAS and ACE2/angiotensin-(1–7)/MAS axis, causing organ injuries. Animal experiments have shown that ACE inhibitor (ACEI) can decrease plasma Ang-II levels and increase the plasma angiotensin (1–7) levels and cardiac ACE2 expression, whereas angiotensin II receptor blockers (ARBs) can increase both Ang-II and angiotensin (1–7) plasma levels as well as ACE2 activity and the cardiac expression.^203^ Thus, the available renin inhibitors, angiotensin (1–7) analogs, and ACEIs/ARBs may relieve organ injuries via the blockage of the renin–angiotensin pathway and/or increased angiotensin-(1–7) levels.^204^ Other animal researches showed that infection with influenza virus in mice or the acute lung injury mediated by SARS-CoV spike could be rescued by ARBs.^205–207^ A population-based study indicated that the ARBs and ACEIs significantly reduced the 30-day mortality rate in patients with pneumonia requiring hospitalization.^208^ Concerns also exist that ACEIs/ARBs treatment may facilitate SARS-CoV infection and increase the risk of severe/fatal COVID-19 progression by enhancing the ACE2 expression levels in target organs.^209^ However, in two large sample studies, ACEIs/ARBs use would not increase SARS-CoV-2 infection.^210^ The prospect of ACEIs/ARBs in COVID-19 treatment needs to be validated in future studies.

#### TMPRSS2

TMPRSS2 is located at 21q22.3 on chromosome 21, and its expression is regulated by androgen signaling through multiple androgen receptor elements upstream of the transcription start site of the gene. Moreover, TMPRSS2 is a protease belonging to the type II transmembrane serine protease family that cleaves the influenza virus hemagglutinin molecule of the human airway epithelial cells.^211^ It can also cleave the S protein, which is activated by protease and induces virus–membrane fusion on the cell surface.^212–215^ The viral hemagglutinin protein binding to ACE2 is the first step in allowing host cell entry. In the second step, hemagglutinin is cleaved, thereby activating internalization. This step depends on the proteases of the host cell, particularly the TMPRSS2.^211^ This highlights the conserved and central role of TMPRSS2 in the pathogenesis of COVID-19. An in vitro study demonstrates that the inhibition of the protease activity of TMPRSS2 partially prevents the entry of SARS-CoV-2 into the lung epithelial cells.^15^ A research conducted by Shutoku et al. demonstrated that TMPRSS2 may be a key protease for SARS-CoV-2 replication and could enhance SARS-CoV-2 infection.^216^ Furthermore, the inhibition of TMPRSS2 activity in the human lung cells by camostat mesylate in vitro was demonstrated to be effective against SARS-CoV-2 infection.^15^ Thus, developing TMPRSS2 inhibitor-associated therapeutic drugs is probably a promising response to the current and new CoVs outbreaks. Moreover, several animal researches indicate that TMPRSS2-knockout mice are protected from disease progression and death after infection with influenza virus.^217,218^ Importantly, in an in vivo study, TMPRSS2-deficient mice were demonstrated to reduce viral replication in the lungs. Furthermore, histopathological and immunohistochemical tests showed that TMPRSS2 expression affected the primary site of infection and the transmission of the virus in the airway with different immunopathologies.^219^ Considering the forceful preclinical support of camostat mesylate for SARS-CoV2 infection, several clinical trials assessing it alone or in combination with HCQ have been initiated in Europe and the United States. Moreover, another TMPRSS2 inhibitor, nafamostat, may be effective against SARS-CoV-2 infection.^220^

Considering the expression of TMPRSS2 that is regulated by androgen signaling, it was found to be highly expressed in the prostate epithelium.^221^ Inhibiting the androgen receptor is an alternative strategy. Before using protease inhibitors or androgen deprivation therapy (ADT) to inhibit the activity of TMPRSS2, understanding the functional polymorphisms of the gene is warranted. Two missense variants (rs12329760; c.589G>A p. Val197Met and rs75603675; c.23G>T p. Gly8Val) within TMPRSS2 have been identified, and their frequencies vary by geography and ancestry. In fact, TMPRSS2 expression on nasal epithelial cells was already found to be higher in Black individuals than in White, Latino, and Asian individuals,^222^ which could explain the 2–3 times higher incidence of COVID-19 in Black individuals than in other individuals.^223^ The functional polymorphisms of TMPRSS2 should be studied as a priority to identify patients who could greatly benefit from these protease inhibitors or ADT.

Although an aberrant fusion of TMPRSS2 with ERG or with other oncogenes, including ETV1, ETV4, and ETV5, is a common trait in prostate cancer,^224^ decreasing the TMPRSS2 expression by inhibition of androgen signaling via use of antiandrogens or ADT that are standard therapies for prostate cancer may be a novel approach against SARS-CoV-2 infection.^225^ Although the safety and effectiveness of these treatments have been well demonstrated in prostate cancer researches,^225^ more preclinical researches are still required to evaluate these novel approaches against SARS-CoV-2 infection.

Serine protease inhibitor might constitute a treatment option through entry blocking^15^ by targeting TMPRSS2. Camostat mesylate, a serine protease inhibitor, was developed in Japan and is applied to treat pancreatitis. Approximately 20 clinical trials on camostat mesylate and COVID-19 are registered in clinicaltrial.gov; however, none of them have been completed. Nafamostat, used as an anticoagulant, is also a serine protease inhibitor.^226^ Japanese scientists disclosed that nafamostat inhibits SARS-CoV-2 in vitro (EC50 = 22.50 µM) by potently binding to TMPRSS2. Additionally, its ability of fusion inhibition is less than one-tenth of the concentration required by camostat.^227^ Thus, nafamostat is also a potential repurposing drug for COVID-19.

### Immunomodulatory factors

Studies have demonstrated a dysregulated immune response in patients with severe COVID-19,^228^ which may be the main cause of lung injury and multiple organ failure. As mentioned above, viral proteins of SARS-CoV-2 have been demonstrated to play important roles in the innate and adaptive immunity. Discovering the characteristics of immune responses to SARS-CoV-2 infection is fundamental for understanding the pathogenesis of COVID-19 and developing immunological therapies. Several methods to modulate the excessive immune response in patients with COVID-19 have been tested in clinical practices.

#### Interferons

IFN, which is a key inflammatory cytokine in CoV infections, is regulated by histone marks, controlling viral infection both in vitro and in vivo.^229^ Moreover, IFN activation is modulated by epigenetic regulators, including H3K4me3, H3K27me3, and H3K9me2.^230,231^ Furthermore, CoVs have ISG effector functions, are actually associated with histone marks of ISG genes at the promoters, and differ from different viruses.^232,233^ IFNs are mainly used in certain kinds of cancers^234^ and hepatitis C.^235^ Researches showed no benefit of IFN-α/β in patients with severe coronavirus (SARS and MERS).^169,236,237^ The early triple combination of IFNβ-1b and lopinavir/ritonavir was preferable to lopinavir–ritonavir alone in negative PCR results, thereby relieving clinical symptoms and shortening hospital stay in patients with mild-to-moderate COVID-19.^155^ Other clinical trials from Iran^238^ and China^239^ have obvious bias that can hardly evaluate the efficacy of IFNs. Conversely, IFNs have obvious adverse events, including flu-like symptoms, headaches, gastrointestinal reactions, and rashes. To data, there is insufficient data to evaluate the potential benefits and toxicity risks of IFNs. Thus, CTGP has not commented on the use of IFNs for patients with mild COVID-19 and recommends against its use in severe or critical COVID-19, except in a clinical trial (AIII).

#### Corticosteroids

Besides inflammatory cytokines, during the cytokine storm, some proinflammatory cytokines (IL- β, -6, -12, -18, and -33 and TNF-α) are always increased in SARS-CoV infection.^240,241^ Moreover, the incidence of cytokine storm is regulated by the demethylation of IFN-regulated and cytokine genes.^242^ Hence, decreasing the plasma level of inflammatory or/and proinflammatory cytokines epigenetically are potential targets to cure COVID-19. Corticosteroid could decrease the severity of cytokine storm and reduce the mortality of patients with SARS-CoV-2 infection.^243^ Dexamethasone is one of the representative drugs of corticosteroids and is mainly used in allergic and autoimmune inflammatory diseases. Based on large, multicenter, randomized, open-label trials, CTGP recommends the use of dexamethasone for certain hospitalized patients with COVID-19.^244–246^ However, this benefit may be offset by adverse effects, including delayed virus clearance^159,247^ and increased risk of secondary infection.^248^ In the RECOVERY trial, the use of dexamethasone significantly reduced the 28-day mortality in patients who needed respiratory support or extra oxygen supply.^249^ The recommendation dose of dexamethasone is 6 mg daily by oral administration or IV injection or dose equivalencies to other corticosteroids. The duration of dexamethasone treatment should be up to 10 days or until hospital discharge. Adverse events, including hyperglycemia, secondary infections, psychiatric effects, and avascular necrosis, should be closely monitored. Additionally, several small-scale clinical trials valuated the efficacy of corticosteroids in COVID-19. In the CoDEX study, compared with the standard of care alone, adding dexamethasone increased the days of survival and free from mechanical ventilation days to >28 days in patients with moderate-to-severe ARDS caused by COVID-19.^245^ However, some studies have different conclusions. A small trial in France showed that hydrocortisone did not reduce mortality or respiratory support in patients with COVID-19 and ARDS in the ICU compared with those with placebo. However, making conclusions is difficult because it was terminated early.^250^ It was noteworthy that, owing to the publication of the RECOVERY study, clinical studies on other corticosteroids were terminated early, resulting in insufficient evaluation of other corticosteroids, including methylprednisolone. However, methylprednisolone has its advantages, including fast-onset time and relatively moderate half-life (12–36 h); thus, it plays an important role in several other diseases with immune disorders in clinical practice. Moreover, in the Metcovid study, methylprednisolone reduced the mortality of patients aged >60 years compared with placebo.^246^ This study has deduced that methylprednisolone has potential in patients with COVID-19 who need corticosteroids. Furthermore, other corticosteroids also have their advantages and disadvantages. Thus, alternative glucocorticoids, including prednisone, methylprednisolone, or hydrocortisone, can be used as well, if dexamethasone is not available.

Theoretically, the pathogenic mechanism of COVID-19 is mainly induced by two processes. In the early stage, the disease is driven by the replication of SARS-CoV-2 and later by excessive inflammatory response. Based on this, it is speculated that antiviral drugs should be collaborated with immunomodulatory therapy in the treatment of COVID-19. The safety and efficacy of a combination therapy of immunoregulatory drugs and antiviral agents for COVID-19 have not been studied in prospective randomized clinical trial. Recently, a preprint article reported the effectiveness of remdesivir with and without dexamethasone in hospitalized patients with COVID-19.^251^ The CTGP recommends the use of dexamethasone plus remdesivir for hospitalized patients with COVID-19 who require extra oxygen supply (BIII). The combination of dexamethasone and remdesivir has a potential, and the optimum time or sequencing of using dexamethasone and remdesivir are worth further studying. Moreover, the combination of corticosteroids and other antiviral drugs are worth assessing.

#### Anti-inflammatory cytokines

Other proinflammatory cytokines or receptor inhibitors, including IL-1 and IL-6 receptor inhibitor, showed significant benefit of survival in patients with COVID-19.^22,252^ The IL-1 inhibitor anakinra is a recombinant human IL-1 receptor antagonist. It was approved for the treatment of rheumatoid arthritis and cryopyrin-associated periodic syndromes.^253,254^ Some case reports reported favorable responses in patients with cytokine release syndrome or macrophage activation syndrome,^255,256^ which were thought to be one of the causes of ARDS among patients with COVID-19. A case–control study in Paris suggested a preferential use of anakinra in patients with severe COVID-19 for reducing the need for invasive mechanical ventilation in the ICU and mortality^257^; however, considering the 14% of patients who died within the first 2 days and 43% of patients who reached the composite primary outcome in the control group, this study had obvious bias. Therefore, the use of IL-1 inhibitors is neither recommended nor contraindicated for treating COVID-19.

Tocilizumab and sarilumab are humanized mAbs against IL-6R, mainly used in rheumatoid arthritis as immunosuppressive drugs.^258,259^ The efficacy and safety of IL-6 inhibitors in patients with COVID-19 have been evaluated^260,261^ and have resulted in some controversial findings. A pilot prospective open, single-arm multicenter study on off-label use of tocilizumab involving 63 hospitalized adult patients with severe COVID-19 demonstrated survival improvement (hazard ratio 2.2 95% confidence interval 1.3–6.7, *p* < 0.05).^262^ Toniati et al. reported that patients with severe COVID-19 with ARDS showed rapid, sustained response to tocilizumab.^263^ However, these studies were limited because no comparison group has been presented. A systematic review and meta-analysis that enrolled 7 retrospective studies involving 592 adult patients with severe COVID-19, including 240 in the tocilizumab group and 352 in the control group, showed nonsignificant differences between the tocilizumab and control groups.^264^ Two large-scale clinical trials, RECOVERY and REMAP-CAP, reported reducing mortality with the use of tocilizumab; however, these trials were also impacted by heterogeneous populations. Because of low-quality evidence, no conclusion has been reached whether tocilizumab should be used in patients with severe COVID-19. But after comprehensive evaluation for the already shown proofs, the CTGP recommended the use of tocilizumab in combination with dexamethasone for hospitalized patients with rapid respiratory decompensation (BIIa); however, siltuximab needs further evaluation owing to insufficient clinical data.

### Important pathways and inhibitors

Because IFN inhibitor can mitigate the inflammation caused by CoV infections, the IFN antagonism therapy is a promising strategy to inhibit SARS-CoV-2 infection as well as the anti-TNF-ɑ antibody therapy, which remarkably dampens IFN content.^241,265^ Another important immune-related pathway, including NF-κB pathway, and its inhibition pathway SIRT1–AMPK signaling pathway as well as the MAPK and Janus kinase (JAK) pathways are also the therapeutic targets, which are involved in immune response and would be regulated by or can influence epigenetic regulation after SARS-CoV-2 infection.^266–269^

#### JAK signal pathway

The JAK signal pathway has been recognized as a key driver of several inflammatory diseases.^270^ Their anti-inflammatory effect makes them a potential target for treating COVID-19. Nowadays, several JAK inhibitors, including baricitinib, ruxolitinib, and tofacitinib, are available. Baricitinib are approved for the treatment of rheumatoid arthritis. In the ACTT-2 study, patients who received baricitinib achieved clinical recovery later than those who received placebo (median recovery time of 7 vs. 8 days), particularly in patients who required high-flow oxygen or noninvasive ventilation, but no statistically significant difference was found in mortality between the two groups.^271^ The side effects of the chronic use of JAK inhibitor are infections, herpes virus reactivation, liver dysfunction, myelosuppression, thrombotic events, and gastrointestinal perforation.^272^ Because of the effect of corticosteroids in severe COVID-19, CTGP recommends baricitinib in combination with remdesivir for the treatment of COVID-19 in hospitalized, nonintubated patients who need extra oxygen supply when corticosteroids is not available (BIIa).

Ruxolitinib is an oral JAK inhibitor targeting JAK1 and JAK2 and has been approved for the treatment of myelofibrosis, erythrocytosis, and acute graft-against-host disease. It inhibits dose-dependent IL-6-induced signal transducer and activator of transcription factor 3 phosphorylation.^273^ A Chinese small-scale randomized clinical trial suggested a radiographic improvement at day 14; however, no difference was observed on discharge time and mortality.^274^ Tofacitinib selectively blocks JAK1 and JAK3 and also has moderate activity on JAK2.^275^ It is approved by the FDA for the treatment of arthritis and ulcerative colitis and is able to reduce IL-6 level in these patients.^276,277^ Owing to the lack of clinical evidence related to COVID-19, the use of JAK inhibitors other than baricitinib to treat COVID-19 is prohibited, except in clinical trials (AIII).

#### Bruton’s tyrosine kinase (BTK)

BTK inhibitors are also considered for use in COVID-19 treatment. BTK, a signaling molecule of cytokine receptor pathways, is important for B cell maturation and function. Acalabrutinib, ibrutinib, and zanubrutinib are representatives of BTK inhibitors that are approved in the treatment of certain lymphomas.^278^ The attempt for use of BTK inhibitors in COVID-19 treatment is limited in small-scale retrospective clinical studies. Mark et al.^279^ found that 10–14 days of acalabrutinib treatment improved the oxygenation of patients with COVID-19 without discernable toxicity. Steven et al.^280^ demonstrated that ibrutinib could prevent lung injury of patients with COVID-19. However, data of these drugs is insufficient to evaluate the efficacy and safety in treating COVID-19.^279,280^ Hence, BTK inhibitors are recommended against COVID-19, except in a clinical trial (AIII).

Although the existing drugs theoretically target the progression of invasion, replication, and release of virus or excessive immune response, only remdesivir, dexamethasone (baricitinib, if dexamethasone cannot be used), and tocilizumab are recommended for use in certain patients with COVID-19 (Table 2). However, their efficacy was unsatisfactory owing to multiple reasons, including low effective concentration, different binding sites, and uncertain mechanism. Our hopes rely on promising potential targets with increasing information on the structure and mechanism of SARS-CoV-2. In the next part, these potential targets will be discussed.

**Table 2:** Repurposing existing drugs for the treatment of COVID-19/SARS-CoV-2 infection

Drug	Current use	Target	Potential target	Recommendation
*Antivirus*				
Antientry				
Umifenovir	Influenza	Prevents fusion of viral and membrane	Nsp7/Nsp8 complex, Nsp14, Nsp15, E-channel, or Spike	Not mentioned
Nelfinavir	HIV	Protease inhibitor	Spike (S) protein	
Aloxistatin	Nervous system disease	Cysteine protease inhibitor	Cathepsin L	
Camostat mesylate	Pancreatitis	Serine protease inhibitor	TMPRSS2	
Nafamostat	Nafamostat			
Chloroquine	Anti-malarial		ACE2, pH, PLpro	Recommend against
Hydroxychloroquine	Autoimmune diseases			
Antireplication				
Protease inhibitor				
lopinavir/ritonavir	HIV	Protease inhibitor	3CLpro	Recommend against (AI)
Darunavir/Cobicistat	HIV		Nsp3c, PLpro, E-channel, Spike proteins	
RNA polymerase inhibitors				
Remdesivir	Ebola virus	RNA-dependent RNA synthetase	Nsp3b, RdRp, E-channel, TMPRSS2	Recommends (BIIa)
Ribavirin	HCV, RSV		PLpro	Not mentioned
Favipiravir	Influenza virus		RdRp	
Anti-release				
Oseltamivir	Influenza A and B	Neuraminidase	3CLpro	Not mentioned
*Immunomodulation*				
Corticosteroids				
Dexamethasone	Allergic or autoimmune disease		Glucocorticoid receptor agonist	Different recommends in different situations
Anti-cytokine interventions				
Anakinra	Rheumatoid arthritis and cryopyrin-associated periodic syndromes	IL-1R		Neither recommend nor against
Tocilizumab	Rheumatoid arthritis	IL-6R		Recommend (BIIa)
Sarilumab				Not mentioned
Kinase inhibitors				
Janus kinase inhibitors				
Baricitinib	Rheumatoid arthritis	JAK1, JAK2, gp130		Recommend in combination with remdesivir if dexamethasone cannot be used (BIIa)
Ruxolitinib	Myelofibrosis	JAK1, JAK2		Recommend against
Tofacitinib	Psoriatic arthritis, juvenile idiopathic arthritis, and ulcerative colitis	JAK1, JAK3		
Bruton’s tyrosine kinase inhibitors				
Acalabrutinib	B cell malignancies	BTK	Immune response of macrophage activation	Recommend against
Ibrutinib	B cell malignancies, chronic graft-vs.-host disease in recipients of stem cell transplantation			
Zanubrutinib	Mantle cell lymphoma			
IFNs	Cancers and hepatitis C			Recommend against
IVIG	Immunoglobulin deficiency, autoimmune diseases		SARS-CoV-2 neutralizing antibodies	Recommend against

*IFN* interferon, *IVIG* intravenous immunoglobulin, *HIV* human immunodeficiency virus, *HCV* hepatitis C virus, *RSV* respiratory fusion virus, *IL* interleukin, *JAK* Janus kinase, *gp* glycoprotein, *BTK* Bruton’s tyrosine kinase, *TMPRSS2* transmembrane protease/serine subfamily member 2, *ACE2* angiotensin-converting enzyme-2, *3CLpro* 3-chymotrypsin-like protease, *RdRp* RNA-dependent RNA polymerase, *PLpro* papain-like cysteine protease, *Nsp* non-structure protein, *pH* potential of hydrogen

## Promising potential targets

In this section, the current state of most promising druggable targets of SARS-CoV-2 was attempted to be summarized based on preclinical categories with the assessment of the advancement of each druggable target. Notably, the targets covered in this section do not include all the potential SARS-CoV-2 targets.

### Spike glycoprotein

#### HR1 and HR2

Fusion inhibitors have been demonstrated to have a significant potential for both prophylaxis and treatment of viral infections. HR1 and HR2 are considered to display typical α-helical structure, which primarily exert their effect on membrane fusion by forming 6-HB. HR1 and HR2 in SARS-CoV-2 exhibits 92.6 and 100% identity with those in SARS-CoV, respectively.^281^ Zhu et al.^282^ analyzed the thermostability and secondary structure of SARS-CoV-2 HR1. They indicated that SARS-CoV-2 HR1 had higher melting temperature (48 vs. 40°C) and α-helical content (66 vs. 41%) than SARS-CoV HR1. Moreover, the binding of HR1 and HR2 showed more stability in SARS-CoV-2 than in SARS-CoV. Cumulatively, a more powerful HR1 and HR2 interaction might exist in SARS-CoV-2, thereby significantly determining its superior fusogenic specialty than SARS-CoV.

Currently, recombinant HR1/HR2 peptides have been reported to block the formation of 6-HB and restrain the fusion of membrane. Existing peptides originated from HR2 include IPB01 and EK1. They could inhibit HR2 to bind with HR1 and thus form 6-HB.^282,283^ Furthermore, several studies have developed a novel recombinant HR2 peptide with the additional attachment of cholesterol groups to carboxy-terminal of HR2, containing IBP02 and EK1C4.^281,282^ The previous evidence that lipid conjugation could enhance antiviral ability and intracorporal stability supports the aforementioned strategies.^284–286^ The resultant lipopeptides are considered to preferentially interact with the cell and virus membranes, therefore improving the inhibitors’ concentrations at the virus fusion site. More studies on compounds targeting HR should be encouraged owing to the wide reactivity displayed in CoV strains.^287^ Most recently, Kandeel et al. assessed that some novel peptides against SARS-CoV-2 fusion by targeting HR2, peptide #2, and its analogs showed their potent inhibition of viral activity and lack of cytotoxicity. These peptides provide an attractive avenue for the development of new therapeutic agents against SARS-CoV-2.^288^ Except for the recombinant peptides, nanoparticle vaccine containing HR has been engineered to evaluate its response in the transgenic hACE2 mice model.^289^ Ma et al. designed a RBD-HR ferritin nanoparticle vaccine and found that it could reduce the substantial number of HR-specific antibodies. Additionally, nAbs elicited by HR antigen could exert positive effects on the cross-protection of other CoVs. Therefore, in the future, HR should be considered to access and develop broad-range vaccines. However, mutations have also been found in this region. Oliva et al.^290^. analyzed 415,673 complete S protein sequences and identified all the mutations occurring on the HR1 fusion core. They found that D936Y is the most frequent mutation in the HR1 fusion core. Further study has demonstrated that the infectivity significantly decreased compared with the Wuhan reference strain^16^ when it was the only variant. However, it has more infectivity when associated with the D614G mutation than the only D614G variant.^290^ Thus, the structural effect of the D936Y variant may still need more researches to identify its potential role in the SARS-CoV-2 virulence. More importantly, long-term monitoring and management of mutations in HR are warranted.

#### Furin cleavage site

The furin cleavage site plays a significant role in the pathogenesis and transmission of SARS-CoV-2. The furin cleavage site is on the S1/S2 boundary of S protein in novel coronavirus, including P681, R682, R683, and A684 (PRRA) four residues.^3^ The polybasic furin cleavage site is unique in SARS-CoV-2 rather than in other CoVs. First, S protein proteolytic activation needs furin proteases, the expression of which omnipresent in human cells. This could result in extensive pathogenesis and tissue tropism^291^ in SARS-CoV-2. Moreover, Johnson et al.^292^ developed a SARS-CoV-2 variant without the furin cleavage spot in the S protein. They found that, compared with the WT virus, the variant decreased the processing and replication of S protein in Vero E6 cells and Calu3 human respiratory cells, respectively. Additionally, Peacock et al. found that the infectivity decreased when SARS-CoV-2 lacked the furin cleavage site and was not transmitted to cohoused sentinel animals compared with the WT virus. Moreover, they identified the selective advantages of the furin cleavage site in the lung and primary human airway epithelial cells depending on the expression of TMPRSS2. These data demonstrated that the furin cleavage site on S protein may play an important role in the high transmissibility and infectivity^293^ of SARS-CoV-2. Further study has demonstrated that the lack of furin cleavage site attenuated pathogenesis of the virus both in hamster and K18-hACE2 transgenic mouse models.^294^ Moreover, this mutation offered protection against rechallenge with the parental virus. Together, these data confirmed the important role of the furin cleavage site in the infection and transmission of SARS-Cov-2 and highlighted the significance of this special region in the development of novel therapeutic strategies against SARS-CoV-2 infection.

### M and E proteins

M and E proteins are both transmembrane glycoproteins containing 220–260 and 76–109 amino acids in SARS-CoV-2, respectively.^295^ The M and E proteins exert important effects in regulating the assembly of the virion. M and E proteins possess sequences of trafficking signal and accumulate in the ER. These proteins efficiently combine with the ribonucleoprotein complex for the budding and maturation of new virion particles.^295^ SARS-CoV-2 M and E proteins share >90% sequence identity with the SARS-CoV homologs.

The current model showed that M protein could interact with S, E, and N proteins to induce membrane curvature during the budding of virion.^296^ Additionally, M protein of the SARS-CoV residues L218 and L219 are essential for N packaging.^297^ It has been reported that M protein could induce strong humoral responses,^298^ apoptosis,^299^ and IFN-β activation.^300^ Liu et al.^298^ has identified the antigenic epitopes of SARS-CoV M protein in the TM region. Therefore, M protein is a potential immunogen in therapy applications. Additionally, Tsoi et al. reported that the C-terminal domain (CTD) of M protein could block the interaction of critical protein kinases (PDK1 and PKB) in impeding the apoptosis process and releasing caspases 8 and 9, ultimately resulting in cell apoptosis and death.^299^ Furthermore, M protein is associated with IFN-β activation by a Toll-like-receptor-related tumor necrosis factor receptor-associated factor 3 (TRAF3). Moreover, Fu et al.^301^ discovered that M protein participates in the innate immune response pathways by interacting with the central adapter proteins MAVS. This interaction attenuated the innate antiviral response through impaired MAVS aggregation and decreased its recruitment of downstream TBK1, TRAF3, and IRF3. These data revealed a mechanism that evades the innate immune response and have demonstrated the potential of M protein as a therapeutic target for the treatment of SARS-CoV-2 infection.

In contrast to M protein, the E protein might also be a promising target for the development of novel agents against SARS-CoV-2. It is the smallest of the major structural proteins and plays critical roles in assembly, budding, and envelope formation of viruses.^302^ Apart from the important role which E protein plays in the replication cycle of SARS-CoV-2, recently, the nuclear magnetic resonance (NMR) structure of E protein in SARS-CoV-2 showed a pentameric helix bundle around a central cationic pore with hydrophilicity.^303,304^ Thus, the E protein could work as the ion-channeling viroporin.^305^ The ion channels result in membrane potential loss and inflammasome activation. Additionally, the interaction of host connection-related proteins (Lin Seven 1/PALS1 and syntenin) and the last four amino acids (DLLV) in E protein might promote the dissemination of virus,^295,306^ which is proposed to be the cause of inducting the cytokine storm together with E protein’s viroporin property. Thus, E protein also majorly affects host inflammation response. It forms a structurally robust but bipartite channel and can interact with drugs, ions, and other viral and host proteins semi-independently through its N- and C-terminal halves based on the NMR structure analysis. Thus, the E inhibitors have been considered optimal antiviral drugs against SARS-CoV-2.^303^ Additionally, recombinant coronavirus without E protein has presented decreased virus titers, damaged virus maturation, and attenuated virus propagation and thus has been expected to be a promising vaccine candidate.^307^ It is noteworthy that Rahman et al. explored only 1.2% mutant strains undergoing complete E protein sequences, highlighting high conservatism (98.8%) of the E protein of SARS-CoV-2.^308^ Their results demonstrated that the E protein evolved slowly compared with other structural proteins. The potential of the E protein has been highlighted as a promising target for both the prophylaxis and treatment of SARS-CoV-2 infection.

### N protein

The N protein is the most abundant viral structural protein in virion or in vivo and is also a strong immunogen.^309,310^ Current evidence indicated that a therapy targeting membraneless organelles or host cell kinases to modulate N protein could be feasible strategies to fight SARS-CoV-2. The N protein is known to be involved in the packaging of the virus. Based on accumulated evidence, Cascarina et al.^311^ proposed that the N protein of SARS-CoV-2 harnesses the capacity of forming or joining biomolecular condensates to disassemble stress granules and improve virus replication or protein translation. Additionally, N protein facilitates virion budding at a proper orientation on the perinuclear, nuclear, endosomal, or plasma membranes, resulting in viral particle release.^312^ Moreover, two druggable sites were found in both NTD and CTD.^313^ In NTD, site 1 included P162, T135, Q83, and Q70-N75. Site 2 included S176, A173, L167, T165, and L159-P162. In CTD, site 1 was located on the central four-stranded β-sheet, whereas site 2 was close to the C-terminal α-helices. However, most recently, Rahman et al.^314^ observed that the N protein of SARS-CoV-2 virus presented higher mutation rate than MERS and SARS-CoV. This situation may challenge the critical role of E protein in the development of vaccines and therapeutics. Therefore, continuous monitoring is required to handle the ongoing mutations of the N protein.

### Papain-like protease

The cysteine proteases encoded by coronaviruses are papain-like protease (PL_pro_, Nsp3).^315,316^ They contribute to the activities of pp1a and pp1ab. The other vital function of PL_pro_ is reducing host immune response power by downregulating crucial signaling molecules such as NF-κB. SARS-CoV-2 PL_pro_ and SARS-CoV PL_pro_ share 83% similarity, whereas the host substrate preferences are different between them: SCoV2-PL_pro_ and SCoV-PL_pro_ mainly cleave the ubiquitin-like ISG15 protein and ubiquitin chain, respectively. The crystal structure showed that SARS-CoV-2 PL_pro_ has high affinity and specificity with ISG15 and modulate the cleavage of ISG15 via combination with IRF3 and reducing type I IFN effects during viral invasion, thus influencing host immune responses (Fig. 4).

Based on the biochemical, structural, and functional studies, new inhibitors against SARS-CoV-2 PL_pro_ have been investigated. Previously, some inhibitors specific against SARS-CoV PL_pro_ were identified. However, none of these inhibitors progressed to clinical usage for SARS-CoV or SARS-CoV-2. Scientists had screened 3727 approved drugs and compounds for repurposing usage in COVID-19 and found no compounds inhibiting PL_pro_ consistently.^317^ A recent study identified seven crystal structures that can recognize specific ligand and interact with PL_pro_ and were proved to inhibit SARS-CoV-2 replication in vitro.^318^ Unfortunately, these drugs have not been tested in vivo or in clinic. Thus, to date, no certain drugs have been found to target PL_pro_ that can be used in COVID-19; however, recent research could provide some insights for further drug designing. Remarkably, SARS-CoV PL_pro_ has been thought to possess IFN-antagonizing activities. Some other Nsps, including SARS-CoV-2 Nsp13, Nsp14, and Nsp15, also showed an ability to inhibit the production of IFN and IFN signaling,^319^ which might also affect immune reaction during the process of SARS-CoV-2 infection.

### Cathepsin L

CatL is considered a promising candidate against SARS-CoV-2 infection. CatL, a key human endosomal cysteine protease, cleaves the virus S1 subunit on spike glycoprotein at an appropriate acidic pH and facilitates the entry of SARS2-CoV2 into the host cell.^15,320^ Compared with healthy individuals, the circulating level of CatL is markedly higher in patients with SARS-CoV-2 infection and is associated with the status and severity of infection. SARS-CoV-2 infection has been found to upregulate CTSL expression and enzyme activity both in vivo and in vitro. In turn, the overexpression and knockdown in vitro and the use of CatL inhibitor in vivo in mice further confirmed the promotion of CatL to ensure coronavirus entry.^321^ Meanwhile, CatL has been demonstrated to not only suppress viral entry but also to interrupt the life cycle of the virus.^321^ Additionally, a majority of available CatL irreversible or reversible inhibitors have been successfully synthesized.^322^ Amantadine, an antivirus drug, is used and licensed to treat influenza. Amantadine markedly suppresses the SARS-CoV-2 via inhibiting the expression and enzyme activity of CatL nearly without cytotoxicity.^321^ X-ray crystal structures of M_pro_ complex showed that the calpain inhibitors II and XII are active against CatL.^322,323^ Heparin has been observed to exert an antiviral response during SARS-CoV-2 infection, which might be associated with impaired S1/S2 proteolytic activity via inhibition of CatL activity.^324^ Teicoplanin can prevent the S protein cleavage by inhibiting CatL activity.^325^ Az peptide nitriles exert strong inhibition toward CatL activity. The combination of M_pro_ and CatL inhibitors is a potent strategy for broadening the therapeutic target spectrum for SARS-CoV-2.^326^ Notably, to date, no drugs are clinically available to treat SARS-CoV-2 infections. The clinical evidence of CatL inhibitors against SARS-CoV-2 infection is lacking. Additionally, the toxicity and unpredictable side effects of CatL inhibitors should be considered owing to the multiple functions of CatL in cells.^327^ In the future, experiments and clinical data are required to validate the use of CatL inhibitors in SARS-CoV-2 infection.

### CD147

Based on the elimination of replication or transcription of viruses and reduced immune effects, CD147 is speculated to be a candidate drug to relieve SARS-CoV-2 infection. CD147 has multiple functions in tumor development, plasmodium invasion, and bacterial and viral infection.^328,329^ CD147 binds to CD147-SP and has been identified as a novel host receptor of SARS-CoV-2 on host cells. Notably, CD147 and ACE2 may be two complementary receptors of SARS-CoV-2.^330^ Conversely, CD147 mediates the increase in the levels of proinflammatory cytokines (i.e., TNF-α, MCP-1, IL-6, and INF-γ), thereby activating immune response widely and inducing tissue damage.^331,332^ Although a Chinese clinical trial in phase II, named “Clinical Study of Anti-CD147 Humanized Meplazumab for Injection to Treat With 2019-nCoV Pneumonia” (ClinicalTrials.gov Identifier: NCT04275245), is currently ongoing to inhibit SARS-CoV-2 S protein binding via suppressing the expression of CD147 protein using Meplazumab,^333^ the main researches targeting CD147 are still in the preclinical stage. Melatonin can not only strongly protect cells from oxidative damage as hydroxyl radical scavenger but also can modulate the immune system by balancing the inflammation and anti-inflammation effects through a CD147-S protein.^334^ Hence, melatonin exerts an antiviral effect by reducing the CD147 levels.^331^ Considering the double immunomodulatory effects of CD147, the use of CD147 suppressor in combination with other antiviral drugs could benefit patients by improving the efficacy of anti-SARS-CoV-2 effect and preventing the potential negative side effects. Remarkably, a novel human CD147 NOD-*scid* IL2Rgamma^null^ (NSG) transgenic mouse model has been successfully developed by Badeti et al.^335^ The hCD147Tg-NSG mouse model may promote the speed of drug development that targets CD147.

### High mobility group box 1

HMGB1 is a highly conserved and multifunctional protein both inside and outside of the cells. In the nucleus, HMGB1 bends DNA as an architectural chromatin-binding factor and regulates DNA replication, transcription, recombination, and repair.^336^ Under stressful conditions, HMGB1 is transferred to the cytoplasm and is secreted extracellularly. Extracellular HMGB1 acts as a crucial member of DAMPs. On the cell surface, HMGB1 binds to classic receptor for advanced glycation end products and Toll-like receptor 2/4/9 and then transmits danger signals to surrounding cells, thereby activating downstream signals and mediating inflammation to infection response.^337,338^ Severe COVID-19 is considered to involve lethal hyperinflammation with cytokine storm syndromes to resist the virus.^22^ HMGB1 plays a vital role in the inflammatory response of COVID-19. The levels of serum HMGB1 in patients with severe COVID-19 obviously increased. The significantly elevated levels of serum HMGB1 correlated with the cytokine storm and high mortality of patients with COVID-19, indicating its potential as a predictor of clinical outcome.^339^ Pathologically, exogenous HMGB1 promotes the expression of ACE2, the SARS-CoV-2 entry receptor, in cultured lung epithelial cells via AGER- or AKT-dependent manner.^340,341^ The regulation of HMGB1 on ACE2 expression is vital for the entry of SARS-CoV-2, SARS-CoV-1, and NL63, thus affecting the susceptibility to SARS-CoV-2.^342^ Thus, HMGB1 is a potential biomarker and therapy target for COVID-19. Based on the changes and direct pathological effects of HMGB1 in COVID-19, genetic inhibitors and pharmacological drugs are explored in the experiments. Genetically, small interfering RNA-mediated depletion of AGER can reduce the HMGB1-induced ACE2 mRNA expression of the lung epithelial cells. Meanwhile, the pharmacological inhibition of HMGB1–AGER pathway limits ACE2 expression in vitro.^340^

Glycyrrhizin, also referred to as glycyrrhizic acid (GLR), is a natural product, mainly isolated from the roots of *Glycyrrhiza glabra* plants. GLR has anti-inflammatory activity against SARS-associated human coronaviruses. At the intracellular and circulating levels, GLR can trap HMGB1 protein and suppress the alarming signals of HMGB1.^343^ Additionally, S-RBD and ORF3a of SARS-CoV-2 can upregulate HMGB1 levels as proinflammatory mediators. GLR can attenuate the expression of S-RBD and ORF3a of SARS-CoV-2 in lung cells. Importantly, GLR can safely inhibit SARS-CoV-2 replication at high doses.^344^ GLR was previously demonstrated as the most active compound in SARS-CoV.^345^ Considering the dual functions of GLR to suppress virus replication and decrease proinflammatory mediators, it should be assessed for use in the treatment of SARS-CoV-2 infection. Moreover, GLR reduces the ACE2 mRNA expression of lung cells in vitro.^340^ These encouraging evidences suggest that HMGB1 inhibitors are similarly promising drug candidates for the treatment of patients with COVID-19.

## Conclusions and perspectives

**Fig. 7:**
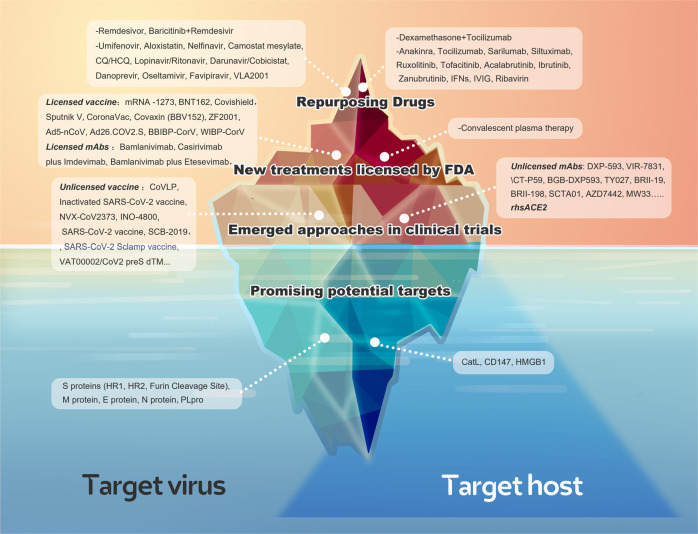
The iceberg model of therapeutic approaches and promising targets against COVID-19 classified by phases of pharmaceutical research

Since the influenza pandemic of 1918, the COVID-19 pandemic becomes the greatest global crisis worldwide. Scientists have been making enormous efforts to understand the pathogenesis of COVID-19 and to find methods to fight against the SARS-CoV-2. First, they focus on the pathogenic viral proteins based on pervious experiences with SARS-CoV and MERS-CoV because they all belong to the same genus and share some common characteristics of viral proteins. Hence, repurposing drugs might be the optimal choice, which can extremely shorten the time of drug development because their efficacy and safety have been already clinically demonstrated. However, the results of repurposing drugs are almost a disappointment. Thus, some important issues have emerged: whether these old drugs are really shortcuts or just distract our attention? Although the main structures of the main viral pathogenic proteins are similar, there is some variation among them: What are these specific differences? Are these differences the key factors that would affect treatment outcomes? Furthermore, how about the regulatory mechanisms of COVID-19 based on host factors? To answer the abovementioned questions, a comprehensive review was further conducted on the current advancements of the emerging interventional strategies and potential targets based on “target virus” and “target host” categories. Regarding SARS-CoV-2, its structural proteins, especially S protein, are still the most promising direct antiviral targets, and the specific details of their crystal structures may play important roles in SARS-CoV-2 replication cycle, even more important than the common structures among the different coronavirus, and may determine the outcomes of the antiviral strategies. Some viral Nsps are vital both in the virus replication and virus–host interactions, which may be indirect targets for the antiviral therapies. Hence, understanding the exact special structures of viral proteins and biological pathogenesis of this disease may reveal novel therapeutic targets against COVID-19. However, with a minor achievement of potential targets/emerging drugs in combating COVID-19, we have to admit that most targets or new drugs were a failure in the preclinical trials. Although a small number of new drugs were privileged to enter clinical trials, majority of them also failed miserably in phase III clinical trials, including vaccines, which should be reconsidered by researchers (Fig. 7). This is the gap between potential targets/new drug discovery and clinical translation. To make a breakthrough in the coming battle with SARS-CoV-2, several approaches should be considered to cover the gap: concentrating on the most potential druggable targets other than casting a wide net in the future drug development, strengthening cooperation among multiple disciplines, and the last but not the least, long-term monitoring of virus mutations are must. The battle between humanity and SARS-CoV-2 has been stalemated for >1 year. Despite tremendous efforts by scientists, there is still a long way to go.

## Supplementary information

Supplement table 1
