# Supplementary material for: Therapeutic targets and interventional strategies in COVID-19: mechanisms and clinical studies
Source: Signal Transduct Target Ther. 2021 Aug 26;6:317. doi: 10.1038/s41392-021-00733-x (PMC8390046; doi:10.1038/s41392-021-00733-x)
Supplement: Supplementary file 1 — Supplement table 1 [file 41392_2021_733_MOESM1_ESM.docx]

**Supplement table 1.** The vaccines against COVID-19 in clinical trials.

| ID | Vaccine platform description | Candidate vaccine code | Type of candidate vaccine | Epitope | Number of doses | Schedule | Route of administration | Developers | The most advanced phase | Phase 1 | Phase 1/2 | Phase 2 | Phase 2/3 | Phase 3 | Phase 4 | Evidence Level | Recommended Level |
| --- | --- | --- | --- | --- | --- | --- | --- | --- | --- | --- | --- | --- | --- | --- | --- | --- | --- |
| 1 | DNA based vaccine | INO-4800 | INO-4800+  electroporation | SP | 2 | Day 0 + 28 | ID | Inovio Pharmaceuticals + International Vaccine Institute + Advaccine (Suzhou) Biopharmaceutical Co., Ltd | Phase 2/3 | [NCT04336410](https://clinicaltrials.gov/ct2/show/NCT04336410) | [NCT04447781](https://clinicaltrials.gov/ct2/show/NCT04447781) | [ChiCTR2000040146](http://www.chictr.org.cn/showprojen.aspx?proj=64452) | [NCT04642638](https://clinicaltrials.gov/ct2/show/NCT04642638) |  |  | Ib | A |
|  |  |  |  |  |  |  |  |  |  | [Study Report](https://www.thelancet.com/journals/eclinm/article/PIIS2589-5370(20)30433-8/fulltext?fbclid=IwAR08A9QJFRt-LNztJ_vmA7uiThT0-BRGc7kFrEU48pxHCQJ21XVkAt7jW5M) |  |  | [Study report](https://www.medrxiv.org/content/10.1101/2021.05.07.21256652v1) |  |  |  |  |
|  |  |  |  |  |  |  |  |  |  | [ChiCTR2000038152](http://www.chictr.org.cn/showproj.aspx?proj=59326) |  |  |  |  |  |  |  |
| 2 |  | AG0301-COVID19 | SP Plasmid DNA Vaccine | SP | 2 | Day 0 + 14 | IM | AnGes + Takara Bio + Osaka University | Phase 2/3 |  | [NCT04463472](https://clinicaltrials.gov/ct2/show/NCT04463472?term=NCT04463472&draw=2&rank=1) |  | [NCT04655625](https://www.clinicaltrials.gov/ct2/show/NCT04655625?term=vaccination&cond=covid&draw=1&rank=129) |  |  |  |  |
|  |  |  |  |  |  |  |  |  |  |  | [NCT04527081](https://clinicaltrials.gov/ct2/show/NCT04527081?term=vaccine&cond=covid-19&draw=7) |  |  |  |  |  |  |
|  |  |  |  |  |  |  |  |  |  |  | [jRCT2051200085](https://jrct.niph.go.jp/en-latest-detail/jRCT2051200085) |  |  |  |  |  |  |
| 3 |  | nCov vaccine | SP Plasmid DNA Vaccine | SP | 3 | Day 0 + 28 + 56 | ID | Zydus Cadila | Phase 3 |  | [CTRI/2020/07/026352](http://ctri.nic.in/Clinicaltrials/pmaindet2.php?trialid=45306&EncHid=&userName=vaccine) |  |  | [CTRI/2020/07/026352](http://ctri.nic.in/Clinicaltrials/showallp.php?mid1=45306&EncHid=&userName=Zydus) |  |  |  |
|  |  |  |  |  |  |  |  |  |  |  | [CTRI/2021/03/032051](http://ctri.nic.in/Clinicaltrials/pmaindet2.php?trialid=53226&EncHid=&userName=covid vaccine) |  |  |  |  |  |  |
| 4 |  | GX-19N | SP Plasmid DNA Vaccine | SP | 2 | Day 0 + 28 | IM | Genexine Consortium | Phase 1/2 |  | [NCT04445389](https://clinicaltrials.gov/ct2/show/NCT04445389?term=vaccine&cond=covid-19&draw=3&rank=12) |  |  |  |  |  |  |
|  |  |  |  |  |  |  |  |  |  |  | [NCT04715997](https://clinicaltrials.gov/ct2/show/NCT04715997) |  |  |  |  |  |  |
| 5 |  | Covigenix VAX-001 | DNA vaccines + proteo-lipid vehicle (PLV) formulation | SP | 2 | Day 0 + 14 | IM | Entos Pharmaceuticals Inc. | Phase 1 | [NCT04591184](https://clinicaltrials.gov/ct2/show/NCT04591184) |  |  |  |  |  |  |  |
| 6 |  | CORVax | SP Plasmid DNA Vaccine | SP | 2 | Day 0 + 14 | ID | Providence Health & Services | Phase 1 | [NCT04627675](https://clinicaltrials.gov/ct2/show/NCT04627675) |  |  |  |  |  |  |  |
| 7 |  | bacTRL | bacTRL-Spike oral DNA vaccine | SP | 1 | Day 0 | Oral | Symvivo Corporation | Phase 1 | [NCT04334980](https://clinicaltrials.gov/ct2/show/NCT04334980) |  |  |  |  |  |  |  |
| 8 |  | GLS-5310 | SP Plasmid DNA Vaccine | SP | 2 | Day 0 + 56 or  Day 0 + 84 | ID | GeneOne Life Science, Inc. | Phase 1/2 |  | [NCT04673149](https://www.clinicaltrials.gov/ct2/results?cond=&term=NCT04673149&cntry=&state=&city=&dist=) |  |  |  |  |  |  |
| 9 |  | COVIGEN | SP Plasmid DNA Vaccine | SP | 2 | Day 0 + 28 | ID or IM | University of Sydney, Bionet Co., Ltd Technovalia | Phase 1 | [NCT04742842](https://clinicaltrials.gov/show/NCT04742842) |  |  |  |  |  |  |  |
| 10 |  | COVID-eVax | SP Plasmid DNA Vaccine | SP | 2 | Day 0 + 28 | IM or IM + electroporation | Takis + Rottapharm Biotech | Phase 1/2 |  | [NCT04788459](https://clinicaltrials.gov/ct2/show/NCT04788459) |  |  |  |  |  |  |
|  |  |  |  |  |  |  |  |  |  |  | [EUCTR2020-003734-20-IT](https://www.clinicaltrialsregister.eu/ctr-search/trial/2020-003734-20/IT) |  |  |  |  |  |  |
| 11 | RNA based vaccine | mRNA -1273 | LNPs–formulated, nucleoside-modified RNA | RBD | 2 | Day 0 + 28 | IM | Moderna + National Institute of Allergy and Infectious Diseases (NIAID) | Phase 4 | [NCT04283461](https://clinicaltrials.gov/ct2/show/NCT04283461?term=vaccine&cond=covid-19&draw=2&rank=4) | [NCT04677660](https://clinicaltrials.gov/ct2/show/NCT04677660) | [NCT04405076](https://clinicaltrials.gov/ct2/show/NCT04405076?term=moderna&cond=covid-19&draw=2&rank=1) | [NCT04649151](https://clinicaltrials.gov/ct2/show/NCT04649151) | [NCT04470427](https://clinicaltrials.gov/ct2/show/NCT04470427?term=vaccine&cond=covid-19&draw=5) | [NCT04760132](https://www.clinicaltrials.gov/ct2/show/NCT04760132?term=vaccine,+phase+4&cond=Covid19&draw=2&rank=1) | Ib | A |
|  |  |  |  |  |  |  |  |  |  | [Interim Report](https://www.nejm.org/doi/full/10.1056/NEJMoa2022483) | [NCT04712110](https://clinicaltrials.gov/ct2/show/NCT04712110) | [Study Report](https://www.sciencedirect.com/science/article/pii/S0264410X21001535) | [NCT04796896](https://www.clinicaltrials.gov/ct2/show/NCT04796896?term=vaccine&type=Intr&cond=Covid19,+sars&strd_s=03/01/2021&strd_e=06/01/2022&draw=2&rank=10) | [Study Report](https://www.nejm.org/doi/10.1056/NEJMoa2035389?url_ver=Z39.88-2003&rfr_id=ori:rid:crossref.org&rfr_dat=cr_pub  0pubmed) | [NCT04792567](https://www.clinicaltrials.gov/ct2/show/NCT04792567?term=vaccine&type=Intr&cond=Covid19,+sars&strd_s=03/01/2021&strd_e=06/01/2022&draw=2&rank=13) |  |  |
|  |  |  |  |  |  |  |  |  |  |  | [NCT04889209](https://www.clinicaltrials.gov/ct2/show/NCT04889209?term=NCT04889209&draw=2&rank=1) |  |  | [NCT04811664](https://www.clinicaltrials.gov/ct2/show/NCT04811664?term=NCT04811664&draw=2&rank=1) |  |  |  |
|  |  |  |  |  |  |  |  |  |  | [Study Report](http://https/www.nejm.org/doi/full/10.1056/NEJMoa2022483) |  | [NCT04761822](https://clinicaltrials.gov/ct2/show/NCT04761822) |  | [NCT04805125](https://clinicaltrials.gov/ct2/show/NCT04805125) |  |  |  |
|  |  |  |  |  |  |  |  |  |  |  |  | [NCT04894435](https://www.clinicaltrials.gov/ct2/show/NCT04894435?term=NCT04894435&draw=2&rank=1) |  | [NCT04806113](https://clinicaltrials.gov/ct2/show/NCT04806113) |  |  |  |
|  |  |  |  |  |  |  |  |  |  | [NCT04839315](https://clinicaltrials.gov/ct2/show/NCT04839315) |  |  |  | [NCT04860297](https://clinicaltrials.gov/ct2/show/NCT04860297) |  |  |  |
| 12 |  | BNT162b2/Comirnaty | 3-LNPs–formulated, nucleoside-modified RNA | SP/RBD | 2 | Day 0 + 21 | IM | Pfizer/BioNTech + Fosun Pharma | Phase 4 | [NCT04523571](https://clinicaltrials.gov/ct2/show/NCT04523571) | [2020-001038-36](https://www.clinicaltrialsregister.eu/ctr-search/search?query=BNT162-01) | [NCT04649021](https://clinicaltrials.gov/ct2/show/NCT04649021) | [NCT04754594](https://clinicaltrials.gov/ct2/show/NCT04754594) | [NCT04368728](https://clinicaltrials.gov/ct2/show/NCT04368728?term=vaccine&cond=covid-19&draw=3&rank=12) | [NCT04760132](https://www.clinicaltrials.gov/ct2/show/NCT04760132?term=vaccine,+phase+4&cond=Covid19&draw=2&rank=1) | Ib | A |
|  |  |  |  |  |  |  |  |  |  | [Study Report](https://www.researchsquare.com/article/rs-137265/v1) | [NCT04588480](https://clinicaltrials.gov/ct2/show/NCT04588480) | [NCT04761822](https://clinicaltrials.gov/ct2/show/NCT04761822) |  | [Study Report](https://www.ncbi.nlm.nih.gov/pmc/articles/PMC7583697/) | [EUCTR2021-000412-28-BE](https://www.clinicaltrialsregister.eu/ctr-search/trial/2021-000412-28/BE) |  |  |
|  |  |  |  |  |  |  |  |  |  | [ChiCTR2000034825](http://www.chictr.org.cn/showprojen.aspx?proj=56834) | [NCT04380701](https://clinicaltrials.gov/ct2/show/NCT04380701) | [NCT04824638](https://clinicaltrials.gov/ct2/show/NCT04824638) |  | [Study Report](https://www.ncbi.nlm.nih.gov/pmc/articles/PMC7745181/) | [EUCTR2021-000412-28-BE](https://www.clinicaltrialsregister.eu/ctr-search/trial/2021-000412-28/BE) |  |  |
|  |  |  |  |  |  |  |  |  |  | [Study Report](https://www.nature.com/articles/s41591-021-01330-9) | [Study Report](https://pubmed.ncbi.nlm.nih.gov/32998157/) | [NCT04860739](https://clinicaltrials.gov/ct2/show/NCT04860739) |  | [NCT04713553](https://clinicaltrials.gov/ct2/show/NCT04713553) | [NCT04780659](https://www.clinicaltrials.gov/ct2/show/NCT04780659?term=vaccine&cond=Covid19&draw=2&rank=14) |  |  |
|  |  |  |  |  |  |  |  |  |  | [NCT04816643](https://clinicaltrials.gov/ct2/show/NCT04816643) | [NCT04537949](https://clinicaltrials.gov/ct2/show/NCT04537949?term=vaccine&cond=covid-19&draw=4&rank=26) | [EUCTR2021-001978-37-ES](https://www.clinicaltrialsregister.eu/ctr-search/trial/2021-001978-37/ES) |  | [NCT04800133](http://https/clinicaltrials.gov/ct2/show/NCT04800133) | [NCT04775069](http://https/clinicaltrials.gov/ct2/show/NCT04775069) |  |  |
|  |  |  |  |  |  |  |  |  |  |  | [EUCTR2020-003267-26-DE](https://www.clinicaltrialsregister.eu/ctr-search/trial/2020-003267-26/DE) | [NCT04894435](https://www.clinicaltrials.gov/ct2/show/NCT04894435?term=NCT04894435&draw=2&rank=1) |  | [NCT04805125](https://clinicaltrials.gov/ct2/show/NCT04805125) | [EUCTR2021-000893-27-BE](https://www.clinicaltrialsregister.eu/ctr-search/trial/2021-000893-27/BE) |  |  |
|  |  |  |  |  |  |  |  |  |  | [NCT04839315](https://clinicaltrials.gov/ct2/show/NCT04839315) | [Study Report](https://www.nature.com/articles/s41586-020-2639-4) |  |  | [NCT04816669](https://clinicaltrials.gov/ct2/show/NCT04816669) | [EUCTR2021-000930-32-BE](https://www.clinicaltrialsregister.eu/ctr-search/trial/2021-000930-32/BE) |  |  |
|  |  |  |  |  |  |  |  |  |  |  | [NCT04889209](https://www.clinicaltrials.gov/ct2/show/NCT04889209?term=NCT04889209&draw=2&rank=1) |  |  |  | [NCT04852861](https://www.clinicaltrials.gov/ct2/show/NCT04852861?term=vaccine&type=Intr&cond=Covid19&draw=2&rank=30) |  |  |
|  |  |  |  |  |  |  |  |  |  |  |  |  |  |  | [NCT04878211](https://clinicaltrials.gov/ct2/show/NCT04878211) |  |  |
| 13 |  | CVnCoV Vaccine | SARS-CoV-2 mRNA vaccine | SP | 2 | Day 0 + 28 | IM | CureVac AG | Phase 3 | [NCT04449276](https://clinicaltrials.gov/ct2/show/NCT04449276?term=vaccine&cond=covid-19&draw=6&rank=47) |  | [NCT04515147](https://clinicaltrials.gov/ct2/show/NCT04515147?term=vaccine&cond=covid-19&draw=11&rank=59) | [NCT04652102](https://www.clinicaltrials.gov/ct2/show/NCT04652102?term=curevac&cond=Covid19&draw=2&rank=1) | [NCT04674189](https://www.clinicaltrials.gov/ct2/show/NCT04674189?id=NCT04639466+OR+NCT04655625+OR+NCT04662697+OR+NCT04683224+OR+NCT04668339+OR+NCT04674189+OR+NCT04665258+OR+NCT04646590+OR+NCT04642638+OR+NCT04656613+OR+NCT04648800+OR+NCT04649515+OR+NCT04677660+OR+NCT04668625+OR+NCT04649021+OR+NCT04649151+OR+NCT04659486+OR+NCT04664075&draw=2&rank=3&load=cart) |  |  |  |
|  |  |  |  |  |  |  |  |  |  |  |  | [PER-054-20](https://www.ins.gob.pe/ensayosclinicos/rpec/recuperarECPBNuevoEN.asp?numec=054-20) |  | [NCT04838847](https://clinicaltrials.gov/ct2/show/NCT04838847) |  |  |  |
|  |  |  |  |  |  |  |  |  |  |  |  |  |  | [NCT04860258](https://clinicaltrials.gov/ct2/show/NCT04860258?term=NCT04860258&draw=2&rank=1) |  |  |  |
| 14 |  | ARCT-021 | ARCT-021 | SP | ND | ND | IM | Arcturus Therapeutics | Phase 2 |  | [NCT04480957](https://clinicaltrials.gov/ct2/show/NCT04480957?term=vaccine&cond=covid-19&draw=10&rank=68) | [NCT04668339](https://www.clinicaltrials.gov/ct2/show/NCT04668339?term=vaccination&cond=covid&draw=1&rank=147) |  |  |  |  |  |
|  |  |  |  |  |  |  |  |  |  |  |  | [NCT04728347](https://clinicaltrials.gov/ct2/show/NCT04728347) |  |  |  |  |  |
| 15 |  | LNP-nCoVsaRNA | LNP-nCoVsaRNA | SP | ND | ND | IM | Imperial College London | Phase 1 | [ISRCTN17072692](http://www.isrctn.com/ISRCTN17072692) |  |  |  |  |  |  |  |
| 16 |  | ARCoV | SARS-CoV-2 mRNA vaccine | RBD | 2 | Day 0 + 14 or  Day 0 + 28 | IM | Academy of Military Science (AMS), Walvax Biotechnology and Suzhou Abogen Biosciences | Phase 3 | [ChiCTR2000034112](http://www.chictr.org.cn/showprojen.aspx?proj=55524) |  | [ChiCTR2100041855](http://www.chictr.org.cn/showprojen.aspx?proj=119719) |  | [NCT04847102](https://clinicaltrials.gov/ct2/show/NCT04847102) |  |  |  |
|  |  |  |  |  |  |  |  |  |  | [ChiCTR2000039212](http://www.chictr.org.cn/showproj.aspx?proj=63183) |  |  |  |  |  |  |  |
| 17 |  | ChulaCov19 | SARS-CoV-2 mRNA vaccine | SP | 2 | Day 0 + 21 | IM | Chulalongkorn University | Phase 1 | [NCT04566276](https://clinicaltrials.gov/ct2/show/NCT04566276) |  |  |  |  |  |  |  |
| 18 |  | PTX-COVID19-B | SARS-CoV-2 mRNA vaccine |  | 2 | Day 0 + 28 | IM | Providence Therapeutics | Phase 1 | [NCT04765436](https://www.clinicaltrials.gov/ct2/show/NCT04765436?id=NCT04747821+OR+NCT04756830+OR+NCT04733807+OR+NCT04728828+OR+NCT04760704+OR+NCT04765436&draw=2&rank=1&load=cart) |  |  |  |  |  |  |  |
| 19 |  | CoV2 SAM (LNP) vaccine | A self-amplifying mRNA (SAM) lipid nanoparticle (LNP) platform + Spike antigen | SP | 2 | Day 0 + 30 | IM | GlaxoSmithKline | Phase 1 | [NCT04758962](https://clinicaltrials.gov/ct2/show/NCT04758962) |  |  |  |  |  |  |  |
| 20 |  | mRNA-1273.351 | A lipid nanoparticle (LNP)-encapsulated mRNA-based vaccine that encodes for a full-length, prefusion stabilized SP of the SARS-CoV-2 B.1.351 variant. | SP | 3 | Day 0 or Day 0 + 28 or Day 56 | IM | Moderna + National Institute of Allergy and Infectious Diseases (NIAID) | Phase 1 | [NCT04785144](https://www.clinicaltrials.gov/ct2/show/NCT04785144?id=NCT04780659+OR+NCT04756830+OR+NCT04773665+OR+NCT04765436+OR+NCT04762680+OR+NCT04785144+OR+NCT04758273+OR+NCT04756323+OR+NCT04761822+OR+NCT04776317+OR+NCT04773067+OR+NCT04750343+OR+NCT04742738+OR+NCT04784767+OR+NCT04760132+OR+NCT04754594+OR+NCT04760743+OR+NCT04760730+OR+NCT04742842+OR+NCT04783311+OR+NCT04748471+OR+NCT04756271+OR+NCT04775069+OR+NCT04764422+OR+NCT04758962+OR+NCT04765384+OR+NCT04743011&draw=2&rank=1&load=cart) |  |  |  |  |  |  |  |
| 21 |  | MRT5500 | SARS-CoV-2 mRNA vaccine | SP | 2 | Day 0 + 21 | IM | Sanofi Pasteur and Translate Bio | Phase 1/2 |  | [NCT04798027](https://www.clinicaltrials.gov/ct2/show/NCT04798027?term=vaccine&type=Intr&cond=Covid19,+sars&strd_s=03/01/2021&strd_e=06/01/2022&draw=2&rank=12) |  |  |  |  |  |  |
| 22 |  | DS-5670a | SARS-CoV-2 mRNA vaccine | SP | 2 |  | IM | Daiichi Sankyo Co., Ltd. | Phase 1/2 |  | [NCT04821674](https://clinicaltrials.gov/ct2/show/NCT04821674) |  |  |  |  |  |  |
| 23 |  | HDT-301 | Self-replicating mRNA vaccine formulated as a lipid nanoparticle. | SP | 2 | Day 0 + 28 | IM | SENAI CIMATEC | Phase 1 | [NCT04844268](https://clinicaltrials.gov/ct2/show/NCT04844268) |  |  |  |  |  |  |  |
| 24 |  | mRNA-1283 | SARS-CoV-2 mRNA vaccine | SP | 2 | Day 0 + 28 | IM | ModernaTX, Inc. | Phase 1 | [NCT04813796](https://clinicaltrials.gov/ct2/show/NCT04813796) |  |  |  |  |  |  |  |
| 25 |  | EXG-5003 | a temperature-sensitive self-replicating RNA vaccine expressing the receptor binding domain of the SARS-CoV-2 SP | RBD | 1 | Day 0 | ID | Elixirgen Therapeutics, Inc | Phase 1/2 |  | [NCT04863131](https://clinicaltrials.gov/ct2/show/NCT04863131?term=vaccine&recrs=abdf&cond=COVID-19&phase=0123&sort=nwst&draw=2) |  |  |  |  |  |  |
| 26 |  | mRNA COVID-19 vaccine | SARS-CoV-2 mRNA vaccine | SP | 2 | TBD | IM | Shanghai East Hospital and Stemirna Therapeutics | Phase 1 | [ChiCTR2100045984](http://www.chictr.org.cn/showprojen.aspx?proj=126046) |  |  |  |  |  |  |  |
| 27 | Viral vector (Non-replicating) | AZD1222/Covishield/Vaxzevria | ChAdOx1-S | SP | 1-2 | Day 0 + 28 | IM | AstraZeneca + University of Oxford | Phase 4 | [PACTR202005681895696](https://pactr.samrc.ac.za/TrialDisplay.aspx?TrialID=10988) | [PACTR202006922165132](https://pactr.samrc.ac.za/TrialDisplay.aspx?TrialID=12166) | [NCT04686773](https://www.clinicaltrials.gov/ct2/show/NCT04686773?id=NCT04639466+OR+NCT04659941+OR+NCT04651790+OR+NCT04659239+OR+NCT04648800+OR+NCT04656613+OR+NCT04672395+OR+NCT04673149+OR+NCT04671017+OR+NCT04685603+OR+NCT04664309+OR+NCT04686773+OR+NCT04681092+OR+NCT04662697+OR+NCT04652102+OR+NCT04665258+OR+NCT04649021+OR+NCT04686409+OR+NCT04666012+OR+NCT04649151+OR+NCT04655625+OR+NCT04684446+OR+NCT04668339+OR+NCT04683224+OR+NCT04674189+OR+NCT04679909&draw=2&rank=1&load=cart) | [NCT04400838](https://clinicaltrials.gov/ct2/show/NCT04400838) | [ISRCTN89951424](http://www.isrctn.com/ISRCTN89951424) | [NCT04760132](https://www.clinicaltrials.gov/ct2/show/NCT04760132?term=vaccine,+phase+4&cond=Covid19&draw=2&rank=1) | Ib | A |
|  |  |  |  |  |  |  |  |  |  |  | [2020-001072-15](https://www.clinicaltrialsregister.eu/ctr-search/trial/2020-001072-15/GB) |  | [Study Report](https://www.thelancet.com/journals/lancet/article/PIIS0140-6736(20)32466-1/fulltext) | [NCT04516746](https://clinicaltrials.gov/ct2/show/NCT04516746?term=astrazeneca&cond=covid-19&draw=2&rank=1) | [NCT04775069](https://clinicaltrials.gov/ct2/show/NCT04775069) |  |  |
|  |  |  |  |  |  |  |  |  |  |  | [Interim Report](https://www.thelancet.com/journals/lancet/article/PIIS0140-6736(20)31604-4/fulltext) | [ISRCTN69254139](https://www.isrctn.com/ISRCTN69254139) | [Study Report](https://papers.ssrn.com/sol3/papers.cfm?abstract_id=3777268) | [NCT04540393](https://clinicaltrials.gov/ct2/show/NCT04540393?term=vaccine&cond=covid-19&draw=3&rank=20) |  |  |  |
|  |  |  |  |  |  |  |  |  |  |  | [NCT04568031](https://clinicaltrials.gov/ct2/show/NCT04568031) |  | [Study Report](https://papers.ssrn.com/sol3/papers.cfm?abstract_id=3779160) | [NCT04536051](https://clinicaltrials.gov/show/NCT04536051) |  |  |  |
|  |  |  |  |  |  |  |  |  |  |  | [Study Report](https://www.thelancet.com/journals/lancet/article/PIIS0140-6736(20)32661-1/fulltext) | [NCT04800133](http://https/clinicaltrials.gov/ct2/show/NCT04800133) | [Study Report](https://www.sciencedirect.com/science/article/pii/S0140673620326611) | [EUCTR2020-005226-28-DE](https://www.clinicaltrialsregister.eu/ctr-search/trial/2020-005226-28/DE) |  |  |  |
|  |  |  |  |  |  |  |  |  |  |  | [NCT04444674](https://clinicaltrials.gov/ct2/show/NCT04444674) | [NCT04860739](https://clinicaltrials.gov/ct2/show/NCT04860739) |  | [Study Report](https://www.thelancet.com/journals/lancet/article/PIIS0140-6736(20)32661-1/fulltext) |  |  |  |
|  |  |  |  |  |  |  |  |  |  |  | [Study Report](https://www.researchsquare.com/article/rs-322470/v1) | [EUCTR2021-001978-37-ES](https://www.clinicaltrialsregister.eu/ctr-search/trial/2021-001978-37/ES) |  |  |  |  |  |
|  |  |  |  |  |  |  |  |  |  |  | [NCT04324606](https://clinicaltrials.gov/ct2/show/NCT04324606) | [NCT04894435](https://www.clinicaltrials.gov/ct2/show/NCT04894435?term=NCT04894435&draw=2&rank=1) | [CTRI/2020/08/027170](http://ctri.nic.in/Clinicaltrials/showallp.php?mid1=46186&EncHid=&userName=covid-19 vaccine) | [Study Report](https://papers.ssrn.com/sol3/papers.cfm?abstract_id=3777268) |  |  |  |
|  |  |  |  |  |  |  |  |  |  |  | [Study Report](https://www.nature.com/articles/s41591-020-01179-4) |  | [ISRCTN69254139](https://www.isrctn.com/ISRCTN69254139) |  |  |  |  |
|  |  |  |  |  |  |  |  |  |  |  | [Study Report](https://pubmed.ncbi.nlm.nih.gov/32702298/) |  | [NCT04885764](https://www.clinicaltrials.gov/ct2/show/NCT04885764?term=NCT04885764&draw=2&rank=1) | [NCT04864561](https://clinicaltrials.gov/ct2/show/NCT04864561) |  |  |  |
|  |  |  |  |  |  |  |  |  |  |  | [Study Report](https://www.nature.com/articles/s41591-020-01194-5) |  |  |  |  |  |  |
|  |  |  |  |  |  |  |  |  |  |  | [NCT04684446](http://https/clinicaltrials.gov/ct2/show/NCT04684446) |  |  |  |  |  |  |
|  |  |  |  |  |  |  |  |  |  |  | [ISRCTN15638344](https://www.isrctn.com/ISRCTN15638344) |  |  |  |  |  |  |
|  |  |  |  |  |  |  |  |  |  |  | [NCT04760730](https://clinicaltrials.gov/ct2/show/NCT04760730) |  |  |  |  |  |  |
|  |  | ChAdOx1 |  |  | 1-2 | Day 0 + 28 | IN | University of Oxford | Phase 1 | [NCT04816019](https://clinicaltrials.gov/ct2/show/NCT04816019) |  |  |  |  |  |  |  |
| 28 |  | Ad5-nCoV | Recombinant Ad5 coronavirus vaccine | SP | 1 | Day 0 | IM | CanSino Biological Inc./Beijing Institute of Biotechnology | Phase 4 | [ChiCTR2000030906](http://www.chictr.org.cn/showprojen.aspx?proj=51154) |  | [ChiCTR2000031781](http://www.chictr.org.cn/showprojen.aspx?proj=52006) |  | [NCT04526990](https://clinicaltrials.gov/ct2/show/NCT04526990?term=vaccine&cond=covid-19&draw=6&rank=48) | [NCT04892459](https://www.clinicaltrials.gov/ct2/show/NCT04892459?term=NCT04892459&draw=2&rank=1) | Ib | A |
|  |  |  |  |  |  |  |  |  |  | [NCT04313127](https://clinicaltrials.gov/ct2/show/NCT04313127) | [NCT04398147](https://clinicaltrials.gov/show/NCT04398147) | [NCT04566770](https://clinicaltrials.gov/ct2/show/NCT04566770) |  | [NCT04540419](https://clinicaltrials.gov/ct2/show/NCT04540419?term=vaccine&cond=covid-19&draw=6) |  |  |  |
|  |  |  |  |  |  |  |  |  |  | [NCT04568811](https://clinicaltrials.gov/ct2/show/NCT04568811) | [NCT04840992](https://clinicaltrials.gov/ct2/show/NCT04840992) | [NCT04341389](https://clinicaltrials.gov/ct2/show/NCT04341389) |  |  |  |  |  |
|  |  |  |  |  |  |  |  |  |  | [NCT04552366](https://clinicaltrials.gov/ct2/show/NCT04552366?term=vaccine&cond=covid-19&draw=3&rank=15) |  |  |  |  |  |  |  |
|  |  |  |  |  |  |  |  |  |  | [Study Report](https://www.thelancet.com/journals/lancet/article/PIIS0140-6736(20)31208-3/fulltext) |  | [Study Report](https://www.thelancet.com/journals/lancet/article/PIIS0140-6736(20)31605-6/fulltext) |  |  |  |  |  |
| 29 |  | Gam-COVID-Vac/ Sputnik V | rAd26-S+rAd5-S | SP | 2 | Day 0 + 21 | IM | Gamaleya Research Institute ; Health Ministry of the Russian Federation | Phase 3 |  | [NCT04436471](https://clinicaltrials.gov/ct2/show/NCT04436471?term=vaccine&cond=covid-19&draw=4) |  |  | [NCT04530396 Study Report](https://clinicaltrials.gov/ct2/show/NCT04530396?term=vaccine&cond=covid-19&draw=3) |  | Ib | A |
|  |  |  |  |  |  |  |  |  |  |  | [NCT04437875](https://clinicaltrials.gov/ct2/show/NCT04437875) |  |  | [NCT04564716](https://clinicaltrials.gov/ct2/show/NCT04564716?term=vaccine&cond=covid-19&draw=3) |  |  |  |
|  |  |  |  |  |  |  |  |  |  |  | [NCT04713488](https://clinicaltrials.gov/ct2/show/NCT04713488) | [NCT04587219](https://clinicaltrials.gov/ct2/show/NCT04587219) |  | [NCT04642339](https://clinicaltrials.gov/show/NCT04642339) |  |  |  |
|  |  |  |  |  |  |  |  |  |  |  | [Study Report](https://www.thelancet.com/journals/lancet/article/PIIS0140-6736(20)31866-3/fulltext) |  | [NCT04640233](https://clinicaltrials.gov/show/NCT04640233) | [NCT04656613](https://www.clinicaltrials.gov/ct2/show/NCT04656613?term=vaccination&cond=covid&draw=2&rank=53) |  |  |  |
|  |  |  |  |  |  |  |  |  |  |  | [NCT04760730](https://clinicaltrials.gov/ct2/show/NCT04760730) |  |  | [NCT04741061](https://www.clinicaltrials.gov/ct2/show/NCT04741061?id=NCT04733807+OR+NCT04718467+OR+NCT04706156+OR+NCT04743947+OR+NCT04715997+OR+NCT04732468+OR+NCT04713488+OR+NCT04741061&draw=2&rank=2&load=cart) |  |  |  |
| 30 |  | Ad26.COV2.S | Ad26-S | SP | 1-2 | Day 0 or Day 0 +56 | IM | Janssen Pharmaceutical | Phase 3 | [NCT04509947](https://clinicaltrials.gov/ct2/show/NCT04509947) | [NCT04436276](https://clinicaltrials.gov/ct2/show/NCT04436276?term=NCT04436276&draw=2&rank=1) | [EUCTR2020-002584-63-DE](https://www.clinicaltrialsregister.eu/ctr-search/search?query=EUCTR2020-002584-63-DE) |  | [NCT04505722](https://clinicaltrials.gov/ct2/show/NCT04505722?term=NCT04505722&draw=2&rank=1) |  | Ib | A |
|  |  |  |  |  |  |  |  |  |  |  | [Study Report](https://www.medrxiv.org/content/10.1101/2020.09.23.20199604v1.full.pdf) | [NCT04535453](https://clinicaltrials.gov/show/NCT04535453) |  | [Study Report](https://www.fda.gov/media/146217/download) |  |  |  |
|  |  |  |  |  |  |  |  |  |  |  |  |  |  | [Study Report](https://www.nejm.org/doi/full/10.1056/NEJMoa2101544?query=featured_home) |  |  |  |
|  |  |  |  |  |  |  |  |  |  |  | [Study Report](https://www.nejm.org/doi/full/10.1056/NEJMoa2034201) | [NCT04765384](https://clinicaltrials.gov/ct2/show/NCT04765384) |  | [NCT04614948](https://clinicaltrials.gov/show/NCT04614948) |  |  |  |
|  |  |  |  |  |  |  |  |  |  |  | [Study Report](https://www.nejm.org/doi/10.1056/NEJMoa2102214) |  |  | [NCT04838795](https://clinicaltrials.gov/ct2/show/NCT04838795) |  |  |  |
|  |  |  |  |  |  |  |  |  |  |  | [NCT04889209](https://www.clinicaltrials.gov/ct2/show/NCT04889209?term=NCT04889209&draw=2&rank=1) |  |  |  |  |  |  |
| 31 |  | GRAd-COV2 | Replication defective Simian Adenovirus (GRAd) encoding SP | SP | 1 | Day 0 | IM | ReiThera + Leukocare + Univercells | Phase 2/3 | [NCT04528641](https://clinicaltrials.gov/ct2/show/NCT04528641?term=vaccine&cond=covid-19&draw=8) |  |  | [NCT04791423](https://clinicaltrials.gov/ct2/show/NCT04791423) |  |  |  |  |
| 32 |  | VXA-CoV2-1 | Ad5 adjuvanted Oral Vaccine platform | SP | 2 | Day 0 + 28 | Oral | Vaxart | Phase 1 | [NCT04563702](https://clinicaltrials.gov/ct2/show/NCT04563702) |  |  |  |  |  |  |  |
| 33 |  | MVA-SARS-2-S | MVA-SARS-2-S | SP | 2 | Day 0 + 28 | IM | University of Munich (Ludwig-Maximilians) | Phase 1 | [NCT04569383](https://clinicaltrials.gov/ct2/show/NCT04569383?term=vaccine&cond=covid-19&draw=5) |  |  |  |  |  |  |  |
| 34 |  | AdCOVID | Adenovirus-based platform expresses the receptor-binding domain (RBD) of the Sars-Cov-2 SP | RBD | 1-2 | Day 0 | IN | Altimmune, Inc. | Phase 1 | [NCT04679909](https://clinicaltrials.gov/ct2/show/NCT04679909) |  |  |  |  |  |  |  |
| 35 |  | BBV154 | Adenoviral vector COVID-19 vaccine | SP | 1 | Day 0 | IN | Bharat Biotech International Limited | Phase 1 | [NCT04751682](https://www.clinicaltrials.gov/ct2/show/NCT04751682?term=vaccine&cond=Coronavirus&sfpd_s=01/01/2021&draw=2&rank=8) |  |  |  |  |  |  |  |
| 36 |  | ChAdV68-S-TCE/SAM-LNP-S-TCE | Chimpanzee Adenovirus serotype 68 (ChAd) and self-amplifying mRNA (SAM) vectors expressing spike alone, or spike plus additional SARS-CoV-2 T cell epitopes. | SP | 2-3 | Day 0 + 14 + 28 or Day 0 +28 + 56 or Day 0 + 112 | IM | Gritstone Oncology | Phase 1 | [NCT04776317](https://clinicaltrials.gov/ct2/show/NCT04776317) |  |  |  |  |  |  |  |
| 37 |  | COVIVAC | Newcastle Disease Virus (NDV) expressing membrane-anchored pre-fusion-stabilized trimeric SARS-CoV-2 S protein +/- adjuvant CpG 1018 | SP | 2 | Day 0 + 28 | IM | Institute of Vaccines and Medical Biologicals, Vietnam | Phase 1/2 |  | [NCT04830800](https://clinicaltrials.gov/ct2/show/NCT04830800) |  |  |  |  |  |  |
| 37 |  | SC-Ad6-1 | Ad6-S | SP | 1-2 | Day 0 +/- 21 | IM | Tetherex Pharmaceuticals Corporation | Phase 1 | [NCT04839042](https://clinicaltrials.gov/ct2/show/NCT04839042) |  |  |  |  |  |  |  |
| 38 |  | MVA-SARS-2-ST | Modified Vaccinia Virus Ankara (MVA) vector expressing a stabilized SARS-CoV-2 SP | SP | 2 | Day 0 + 28 | IM | German Center for Infection Research | Phase 1/2 |  | [NCT04895449](https://www.clinicaltrials.gov/ct2/show/NCT04895449?term=NCT04895449&draw=2&rank=1) |  |  |  |  |  |  |
| 39 |  | hAd5-S-Fusion+N-ETSD vaccine | Human Adenovirus Type 5: hAd5 S+N bivalent vaccine (S-Fusion + N-ETSD). E2b- Deleted Adeno. | SP | 1-2 | Day 0 + 21 | SC or Oral or SL | ImmunityBio, Inc | Phase 1/2 | [NCT04591717](https://clinicaltrials.gov/ct2/show/NCT04591717) | [NCT04843722](https://clinicaltrials.gov/ct2/show/NCT04843722) |  |  |  |  |  |  |
|  |  |  |  |  |  |  |  |  |  | [NCT04710303](https://www.clinicaltrials.gov/ct2/show/NCT04710303?id=NCT04639466+OR+NCT04656613+OR+NCT04649021+OR+NCT04649151+OR+NCT04655625+OR+NCT04691947+OR+NCT04706390+OR+NCT04706143+OR+NCT04695652+OR+NCT04694651+OR+NCT04710303+OR+NCT04706156+OR+NCT04691908+OR+NCT04672395+OR+NCT04709003+OR+NCT04685603+OR+NCT04673149+OR+NCT04671017+OR+NCT04686773+OR+NCT04681092+OR+NCT04674189+OR+NCT04690387+OR+NCT04684446+OR+NCT04668339+OR+NCT04702295+OR+NCT04683224&draw=2&rank=1&load=cart) | [NCT04845191](https://clinicaltrials.gov/ct2/show/NCT04845191) |  |  |  |  |  |  |
|  |  |  |  |  |  |  |  |  |  | [NCT04732468](https://clinicaltrials.gov/ct2/show/NCT04732468) | [NCT04843722](https://clinicaltrials.gov/ct2/show/NCT04843722) |  |  |  |  |  |  |
| 40 |  | COH04S1 | Modified vaccinia ankara (sMVA) platform + synthetic SARS-CoV-2 | SP | 1-2 | Day 0 + 28 | IM | City of Hope Medical Center + National Cancer Institute | Phase 1 | [NCT04639466](https://clinicaltrials.gov/ct2/show/NCT04639466) |  |  |  |  |  |  |  |
| 41 | Viral vector (Non-replicating) + APC | LV-SMENP-DC | Dendritic cells are modified with lentivirus vectors expressing Covid-19 minigene SMENP and immune modulatory genes. CTLs are activated by LV-DC presenting Covid-19 specific antigens. | SP | 1 | Day 0 | SC & IV | Shenzhen Geno-Immune Medical Institute | Phase 1/2 |  | [NCT04276896](https://clinicaltrials.gov/ct2/show/NCT04276896) |  |  |  |  |  |  |
| 42 | Viral vector (Replicating) | DelNS1-2019-nCoV-RBD-OPT1 | Intranasal flu-based-RBD | RBD | 2 | Day 0 + 28 | IN | University of Hong Kong, Xiamen University and Beijing Wantai Biological Pharmacy | Phase 2 | [ChiCTR2000037782](http://www.chictr.org.cn/showprojen.aspx?proj=55421) |  | [ChiCTR2000039715](http://www.chictr.org.cn/showproj.aspx?proj=63754) |  |  |  |  |  |
|  |  |  |  |  |  |  |  |  |  | [NCT04809389](https://clinicaltrials.gov/ct2/show/NCT04809389) |  |  |  |  |  |  |  |
| 43 |  | rVSV-SARS-CoV-2-S Vaccine | rVSV-SARS-CoV-2-S | SP | 1 | Day 0 | IM | Israel Institute for Biological Research | Phase 1/2 |  | [NCT04608305](https://clinicaltrials.gov/show/NCT04608305) |  |  |  |  |  |  |
| 44 |  | AdCLD-CoV19 | Adenovirus vector | SP | 1 | Day 0 | IM | Cellid Co., Ltd. | Phase 1/2 |  | [NCT04666012](https://clinicaltrials.gov/ct2/show/NCT04666012) |  |  |  |  |  |  |
| 45 | Viral vector (Replicating) + APC | Covid-19/aAPC vaccine | The Covid-19/aAPC vaccine is prepared by applying lentivirus modification with immune modulatory genes and the viral minigenes to the artificial antigen presenting cells (aAPCs). | SP | 3 | Day 0 + 14 + 28 | SC | Shenzhen Geno-Immune Medical Institute | Phase 1 | [NCT04299724](https://clinicaltrials.gov/ct2/show/study/NCT04299724) |  |  |  |  |  |  |  |
| 46 |  | Dendritic cell vaccine AV-COVID-19 | A vaccine consisting of autologous dendritic cells loaded with antigens from SARS-CoV-2, with or without GM-CSF | SP | 1 | Day 0 | IM | Aivita Biomedical, Inc.  National Institute of Health Research and Development, Ministry of Health Republic of Indonesia | Phase 1/2 | [NCT04690387](https://www.clinicaltrials.gov/ct2/show/NCT04690387?id=NCT04639466+OR+NCT04659941+OR+NCT04691947+OR+NCT04651790+OR+NCT04659239+OR+NCT04648800+OR+NCT04691908+OR+NCT04656613+OR+NCT04672395+OR+NCT04673149+OR+NCT04671017+OR+NCT04685603+OR+NCT04664309+OR+NCT04686773+OR+NCT04681092+OR+NCT04662697+OR+NCT04652102+OR+NCT04665258+OR+NCT04649021+OR+NCT04686409+OR+NCT04690387+OR+NCT04666012+OR+NCT04649151+OR+NCT04655625+OR+NCT04684446+OR+NCT04668339+OR+NCT04683224+OR+NCT04674189+OR+NCT04690816+OR+NCT04679909&draw=2&rank=4&load=cart) | [NCT04386252](https://clinicaltrials.gov/ct2/show/NCT04386252) |  |  |  |  |  |  |
|  |  |  |  |  |  |  |  |  |  | [NCT04685603](https://www.clinicaltrials.gov/ct2/show/NCT04685603?id=NCT04639466+OR+NCT04659941+OR+NCT04651790+OR+NCT04659239+OR+NCT04648800+OR+NCT04656613+OR+NCT04672395+OR+NCT04673149+OR+NCT04671017+OR+NCT04685603+OR+NCT04664309+OR+NCT04686773+OR+NCT04681092+OR+NCT04662697+OR+NCT04652102+OR+NCT04665258+OR+NCT04649021+OR+NCT04686409+OR+NCT04666012+OR+NCT04649151+OR+NCT04655625+OR+NCT04684446+OR+NCT04668339+OR+NCT04683224+OR+NCT04674189+OR+NCT04679909&draw=2&rank=3&load=cart) |  |  |  |  |  |  |  |
| 47 | Inactivated virus | CoronaVac | inactivated SARS-CoV-2 vaccine (vero cell) | Viral protein | 2 | Day 0 + 14 | IM | Sinovac Research and Development Co., Ltd | Phase 4 |  | [NCT04383574](https://clinicaltrials.gov/ct2/show/NCT04383574?term=covid-19&cond=vaccine&cntry=CN&draw=2&rank=3) | [NCT04800133](https://www.clinicaltrials.gov/ct2/show/NCT04800133?term=NCT04800133&draw=2&rank=1) |  | [NCT04456595](https://clinicaltrials.gov/ct2/show/NCT04456595?term=vaccine&cond=covid-19&draw=2&rank=1) | [NCT04756830](http://https/clinicaltrials.gov/ct2/show/NCT04756830) | Ib | A |
|  |  |  |  |  |  |  |  |  |  |  |  | [NCT04884685](https://www.clinicaltrials.gov/ct2/show/NCT04884685?term=NCT04884685&draw=2&rank=1) |  | [Study Report](https://papers.ssrn.com/sol3/papers.cfm?abstract_id=3822780) | [NCT04747821](https://clinicaltrials.gov/ct2/show/NCT04747821) |  |  |
|  |  |  |  |  |  |  |  |  |  |  | [Study Report](https://pubmed.ncbi.nlm.nih.gov/33548194/) |  |  | [Study Protocol](https://pubmed.ncbi.nlm.nih.gov/33059771/) | [NCT04775069](https://clinicaltrials.gov/ct2/show/NCT04775069) |  |  |
|  |  |  |  |  |  |  |  |  |  |  | [NCT04352608](https://clinicaltrials.gov/ct2/show/NCT04352608?term=Sinovac&cntry=CN&draw=2&rank=9https://clinicaltrials.gov/ct2/show/NCT04352608?term=Sinovac&cntry=CN&draw=2&rank=9) |  |  | [NCT04508075](https://clinicaltrials.gov/ct2/show/NCT04508075) | [NCT04789356](https://clinicaltrials.gov/ct2/show/NCT04789356) |  |  |
|  |  |  |  |  |  |  |  |  |  |  | [Study Report](https://www.thelancet.com/journals/laninf/article/PIIS1473-3099(20)30843-4/fulltext) |  |  |  | [NCT04754698](http://http/www.chictr.org.cn/showproj.aspx?proj=63353) |  |  |
|  |  |  |  |  |  |  |  |  |  |  | [NCT04551547](https://clinicaltrials.gov/ct2/show/NCT04551547?term=vaccine&cond=covid-19&draw=2&rank=8) |  |  | [NCT04582344](https://clinicaltrials.gov/ct2/show/NCT04582344?term=sinovac++vaccine&cond=covid&draw=2&rank=5) | [NCT04801888](https://clinicaltrials.gov/ct2/show/NCT04801888) |  |  |
|  |  |  |  |  |  |  |  |  |  |  | [Study Report](https://papers.ssrn.com/sol3/papers.cfm?abstract_id=3820545) |  |  | [NCT04617483](https://clinicaltrials.gov/ct2/show/NCT04617483?term=sinovac++vaccine&cond=covid&draw=2&rank=7) | [NCT04894227](https://www.clinicaltrials.gov/ct2/show/NCT04894227?term=NCT04894227&draw=2&rank=1) |  |  |
|  |  |  |  |  |  |  |  |  |  |  |  |  |  | [NCT04651790](https://www.clinicaltrials.gov/ct2/show/NCT04651790?term=vaccination&cond=covid&draw=2&rank=10) | [NCT04892459](https://www.clinicaltrials.gov/ct2/show/NCT04892459?term=NCT04892459&draw=2&rank=1) |  |  |
|  |  |  |  |  |  |  |  |  |  |  |  |  |  | [Study Report](https://www.medrxiv.org/content/10.1101/2021.03.31.21254494v1) |  |  |  |
| 48 |  | WIBP-CorV | Inactivated SARS-CoV-2 vaccine (Vero cell) | Viral protein | 2 | Day 0 + 21 | IM | Sinopharm + China National Biotec Group Co + Wuhan Institute of Biological Products | Phase 3 |  | [ChiCTR2000031809](http://www.chictr.org.cn/showprojen.aspx?proj=52227) |  | [NCT04885764](https://www.clinicaltrials.gov/ct2/show/NCT04885764?term=NCT04885764&draw=2&rank=1) | [ChiCTR2000034780](http://www.chictr.org.cn/showprojen.aspx?proj=56651) |  | Ib | A |
|  |  |  |  |  |  |  |  |  |  |  |  |  |  | [ChiCTR2000039000](http://www.chictr.org.cn/showprojen.aspx?proj=62581) |  |  |  |
|  |  |  |  |  |  |  |  |  |  |  |  |  |  | [NCT04510207 *](https://clinicaltrials.gov/ct2/show/NCT04510207) |  |  |  |
|  |  |  |  |  |  |  |  |  |  |  | [Interim Report](https://jamanetwork.com/journals/jama/fullarticle/2769612) |  |  | [NCT04612972](https://clinicaltrials.gov/ct2/show/NCT04612972) |  |  |  |
| 49 |  | BBIBP-CorV | Inactivated SARS-CoV-2 vaccine (Vero cell) | Viral protein | 2 | Day 0 + 21 | IM | Sinopharm + China National Biotec Group Co + Beijing Institute of Biological Products | Phase 3 |  | [ChiCTR2000032459](http://www.chictr.org.cn/showproj.aspx?proj=53003) |  |  | [NCT04560881](https://clinicaltrials.gov/ct2/show/NCT04560881?term=vaccine&cond=covid-19&draw=2&rank=3) | [NCT04863638](https://clinicaltrials.gov/ct2/show/NCT04863638) | Ib | A |
|  |  |  |  |  |  |  |  |  |  |  |  |  |  |  |  |  |  |
|  |  |  |  |  |  |  |  |  |  |  | [Study Report](https://www.thelancet.com/journals/laninf/article/PIIS1473-3099(20)30831-8/fulltext) |  |  | [NCT04510207*](https://clinicaltrials.gov/ct2/show/NCT04510207) |  |  |  |
| 50 |  | SARS-CoV-2 vaccine (vero cells) | SARS-CoV-2 vaccine (vero cells) | Viral protein | 2 | Day 0 + 28 | IM | Institute of Medical Biology + Chinese Academy of Medical Sciences | Phase 3 |  | [NCT04470609](https://clinicaltrials.gov/ct2/show/NCT04470609?term=vaccine&cond=covid-19&draw=2) |  |  | [NCT04659239](https://www.clinicaltrials.gov/ct2/show/NCT04659239?term=vaccination&cond=covid&draw=3&rank=19) |  | Ib | A |
|  |  |  |  |  |  |  |  |  |  |  | [NCT04412538](https://clinicaltrials.gov/ct2/show/NCT04412538?term=vaccine&cond=covid-19&draw=2) |  |  |  |  |  |  |
|  |  |  |  |  |  |  |  |  |  |  | [Study Report](https://www.medrxiv.org/content/10.1101/2020.09.27.20189548v1) |  |  |  |  |  |  |
|  |  |  |  |  |  |  |  |  |  |  | [Study Report](https://www.sciencedirect.com/science/article/pii/S0264410X2100431X?via=ihub) |  |  |  |  |  |  |
| 51 |  | QazCovid-in® | COVID-19 inactivated vaccine | Viral protein | 2 | Day 0 + 21 | IM | Research Institute for Biological Safety Problems, Rep of Kazakhstan | Phase 3 |  | [NCT04530357](https://clinicaltrials.gov/ct2/show/NCT04530357?term=vaccine&cond=covid-19&draw=4) |  |  | [NCT04691908](https://www.clinicaltrials.gov/ct2/show/NCT04691908?id=NCT04639466+OR+NCT04659941+OR+NCT04691947+OR+NCT04651790+OR+NCT04659239+OR+NCT04648800+OR+NCT04691908+OR+NCT04656613+OR+NCT04672395+OR+NCT04673149+OR+NCT04671017+OR+NCT04685603+OR+NCT04664309+OR+NCT04686773+OR+NCT04681092+OR+NCT04662697+OR+NCT04652102+OR+NCT04665258+OR+NCT04649021+OR+NCT04686409+OR+NCT04690387+OR+NCT04666012+OR+NCT04649151+OR+NCT04655625+OR+NCT04684446+OR+NCT04668339+OR+NCT04683224+OR+NCT04674189+OR+NCT04690816+OR+NCT04679909&draw=2&rank=2&load=cart) |  |  |  |
| 52 |  | BBV152/Covaxin | Whole-Virion Inactivated SARS-CoV-2 Vaccine | Viral protein | 2 | Day 0 + 14 | IM | Bharat Biotech International Limited | Phase 3 |  | [NCT04471519](https://clinicaltrials.gov/ct2/show/NCT04471519?term=bharat&cond=covid-19&draw=2&rank=1) |  |  | [NCT04641481; CTRI/2020/11/028976](https://clinicaltrials.gov/ct2/show/NCT04641481) |  | Ib | A |
|  |  |  |  |  |  |  |  |  |  |  | [Interim Study Report](https://www.medrxiv.org/content/10.1101/2020.12.11.20210419v1) |  |  |  |  |  |  |
|  |  |  |  |  |  |  |  |  |  |  | [Study Report](https://www.medrxiv.org/content/10.1101/2020.12.21.20248643v1) |  |  |  |  |  |  |
|  |  |  |  |  |  |  |  |  |  |  | [Study Report](https://www.thelancet.com/journals/laninf/article/PIIS1473-3099(20)30942-7/fulltext) |  |  |  |  |  |  |
|  |  |  |  |  |  |  |  |  |  |  | [Study report](https://www.thelancet.com/journals/laninf/article/PIIS1473-3099(21)00070-0/fulltext) |  |  |  |  |  |  |
|  |  |  |  |  |  |  |  |  |  |  | [CTRI/2020/07/026300](http://ctri.nic.in/Clinicaltrials/showallp.php?mid1=45184&EncHid=&userName=bbv152) |  |  |  |  |  |  |
|  |  |  |  |  |  |  |  |  |  |  | [CTRI/2020/09/027674](http://ctri.nic.in/Clinicaltrials/pmaindet2.php?trialid=46312&EncHid=&userName=vaccine) |  |  |  |  |  |  |
| 53 |  | Inactivated SARS-CoV-2 vaccine (Vero cell) | Inactivated SARS-CoV-2 vaccine (Vero cell) | Viral protein | 1,2 or 3 | ND | IM | Beijing Minhai Biotechnology Co | Phase 3 | [NCT04758273](http://https/clinicaltrials.gov/ct2/show/NCT04758273) |  | [NCT04756323](http://www.chictr.org.cn/showproj.aspx?proj=63353) |  | [NCT04852705](https://clinicaltrials.gov/ct2/show/NCT04852705?term=vaccine&recrs=abdf&cond=COVID-19&phase=0123&sort=nwst&draw=2&rank=1) |  | Ib | A |
|  |  |  |  |  |  |  |  |  |  | [Study Report](https://www.medrxiv.org/content/10.1101/2021.04.07.21253850v1) |  | [Study Report](https://www.medrxiv.org/content/10.1101/2021.04.07.21253850v1) |  |  |  |  |  |
| 54 |  | VLA2001 | Inactiviated Adjuvanted SARS-CoV-2 Virus Vaccine | Viral protein | 2 | Day 0 + 21 | IM | Valneva, National Institute for Health Research, United Kingdom | Phase 3 |  | [NCT04671017](https://clinicaltrials.gov/ct2/show/NCT04671017?lead=Valneva&draw=2&rank=1) |  |  | [NCT04864561](https://clinicaltrials.gov/ct2/show/NCT04864561) |  |  |  |
| 55 |  | ERUCOV-VAC | Inactivated virus | Viral protein | 2 | Day 0 + 21 | IM | Erciyes University | Phase 2 | [NCT04691947](https://www.clinicaltrials.gov/ct2/show/NCT04691947?id=NCT04639466+OR+NCT04659941+OR+NCT04691947+OR+NCT04651790+OR+NCT04659239+OR+NCT04648800+OR+NCT04691908+OR+NCT04656613+OR+NCT04672395+OR+NCT04673149+OR+NCT04671017+OR+NCT04685603+OR+NCT04664309+OR+NCT04686773+OR+NCT04681092+OR+NCT04662697+OR+NCT04652102+OR+NCT04665258+OR+NCT04649021+OR+NCT04686409+OR+NCT04690387+OR+NCT04666012+OR+NCT04649151+OR+NCT04655625+OR+NCT04684446+OR+NCT04668339+OR+NCT04683224+OR+NCT04674189+OR+NCT04690816+OR+NCT04679909&draw=2&rank=1&load=cart) |  | [NCT04824391](https://clinicaltrials.gov/ct2/show/NCT04824391) |  |  |  |  |  |
| 56 |  | Shif-Pharmed | COVID-19 inactivated vaccine | Viral protein | 2 | Day 0 + 14 | IM | Shifa Pharmed Industrial Co | Phase 2/3 | [IRCT20201202049567N1](https://en.irct.ir/trial/52701) |  |  | [IRCT20201202049567N3](https://en.irct.ir/trial/54881) |  |  |  |  |
|  |  |  |  |  |  |  |  |  |  | [IRCT20201202049567N2](https://en.irct.ir/trial/54885) |  |  |  |  |  |  |  |
| 57 |  | NDV-HXP-S | Inactivated (NDV-based) chimeric vaccine with or without the adjuvant CpG 1018 | Viral protein | 2 | Day 0 + 28 | IM | The Government Pharmaceutical Organization (GPO); PATH; Dynavax | Phase 1/2 |  | [NCT04764422](https://clinicaltrials.gov/ct2/show/NCT04764422) |  |  |  |  |  |  |
| 58 |  | MIVAC | Inactivated SARS-CoV-2 vaccine FAKHRAVAC | Viral protein | 2 | Day 0 + 14 +/- 21 | IM | Organization of Defensive Innovation and Research | Phase 1 | [IRCT20210206050259N1](https://en.irct.ir/trial/54133) |  |  |  |  |  |  |  |
| 59 |  | Koçak-19 Inaktif Adjuvanlı | Inactivated COVID-19 vaccine | Viral protein | 2 | Day 0 + 21 | IM | Kocak Farma | Phase 1 | [NCT04838080](https://www.clinicaltrials.gov/ct2/show/NCT04838080?term=NCT04838080&draw=2&rank=1) |  |  |  |  |  |  |  |
| 60 |  | SARS-CoV-2 adjuvanted inactivated vaccine | Adjuvanted inactivated vaccine against SARS-CoV-2 | Viral protein | 2 | Day 0 + 21 | SC | The Scientific and Technological Research Council of Turkey (TÜBITAK) | Phase 1 | [NCT04866069](https://www.clinicaltrials.gov/ct2/show/NCT04866069) |  |  |  |  |  |  |  |
| 61 |  | KD-414 | Inactivated COVID-19 vaccine | Viral protein | 2 | Day 0 + 28 | IM | KM Biologics Co., Ltd. | Phase 1/2 |  | [jRCT2071200106](https://jrct.niph.go.jp/en-latest-detail/jRCT2071200106) |  |  |  |  |  |  |
| 62 |  | Live rNDV Based Vaccine | Live recombinant Newcastle Disease Virus (rNDV) vector vaccine | Viral protein | 2 | Day 0 + 21 | IM or IN | Laboratorio Avi-Mex | Phase 1 | [NCT04871737](https://clinicaltrials.gov/ct2/show/NCT04871737) |  |  |  |  |  |  |  |
| 63 | Live attenuated virus | COVI-VAC | Live Attenuated Vaccine | Viral protein | 1-2 | Day 0 or  Day 0 + 28 | IN | Codagenix/Serum Institute of India | Phase 1 | [NCT04619628](https://clinicaltrials.gov/ct2/show/NCT04619628) |  |  |  |  |  |  |  |
| 64 |  | MV-014-212 | Live attenuated vaccine that expresses the SP of SARS-CoV-2 | SP | 3 | Day 0 +/- 35 | IN | Meissa Vaccines, Inc. | Phase 1 | [NCT04798001](https://clinicaltrials.gov/ct2/show/NCT04798001?term=covid-19+vaccine&draw=2) |  |  |  |  |  |  |  |
| 65 | Protein subunit | NVX-CoV2373 | SARS-CoV-2 rS/Matrix M1-Adjuvant (Full length recombinant SARS CoV-2 glycoprotein nanoparticle vaccine adjuvanted with Matrix M) | SP | 2 | Day 0 + 21 | IM | Novavax | Phase 3 |  | [NCT04368988](https://clinicaltrials.gov/ct2/show/NCT04368988?term=vaccine&recrs=a&cond=covid-19&draw=2&rank=10) | [NCT04533399](https://clinicaltrials.gov/ct2/show/NCT04533399?term=vaccine&cond=covid-19&draw=7) |  | [NCT04611802](https://clinicaltrials.gov/ct2/show/NCT04611802?term=NCT04611802&draw=2&rank=1) |  | Ib | A |
|  |  |  |  |  |  |  |  |  |  |  | [Study Report](https://www.nejm.org/doi/full/10.1056/NEJMoa2026920?query=featured_home) | [Study Report](https://www.nejm.org/doi/10.1056/NEJMoa2103055) |  | [EUCTR2020-004123-16-GB](https://www.clinicaltrialsregister.eu/ctr-search/search?query=EUCTR2020-004123-16-GB) |  |  |  |
|  |  |  |  |  |  |  |  |  |  |  | [Study Report](https://pubmed.ncbi.nlm.nih.gov/33139139/) |  |  | [NCT04583995](https://clinicaltrials.gov/ct2/show/NCT04583995) |  |  |  |
| 66 |  | ZF2001 | Recombinant SARS-CoV-2 vaccine (CHO Cell) | RBD | 2-3 | Day 0 + 28 or Day 0 + 28 + 56 | IM | Anhui Zhifei Longcom Biopharmaceutical + Institute of Microbiology, Chinese Academy of Sciences | Phase 3 | [NCT04445194 Study Report](https://clinicaltrials.gov/ct2/show/NCT04445194?term=longcom&draw=2&rank=2) | [NCT04550351](https://clinicaltrials.gov/ct2/show/NCT04550351?term=vaccine&cond=covid-19&draw=13&rank=114) | [NCT04466085](https://clinicaltrials.gov/ct2/show/NCT04466085?term=NCT04466085&draw=2&rank=1) |  |  |  | Ib | A |
|  |  |  |  |  |  |  |  |  |  | [ChiCTR2000035691](http://www.chictr.org.cn/showproj.aspx?proj=58207) |  | [Study Report](https://www.thelancet.com/journals/laninf/article/PIIS1473-3099(21)00127-4/fulltext) |  | [NCT04646590](https://clinicaltrials.gov/ct2/show/NCT04646590) |  |  |  |
|  |  |  |  |  |  |  |  |  |  | [NCT04636333](https://clinicaltrials.gov/ct2/show/NCT04636333) | [NCT04813562](https://clinicaltrials.gov/ct2/show/NCT04813562) |  |  | [Study Report](https://www.thelancet.com/journals/laninf/article/PIIS1473-3099(21)00127-4/fulltext) |  |  |  |
| 67 |  | KBP-COVID-19 | RBD-based vaccine | RBD | 2 | Day 0 + 21 | IM | Kentucky Bioprocessing Inc. | Phase 1/2 |  | [NCT04473690](https://clinicaltrials.gov/ct2/show/study/NCT04473690?term=vaccine&cond=covid-19&draw=3) |  |  |  |  |  |  |
| 68 |  | VAT00002/CoV2 preS dTM | SARS-CoV-2 vaccine formulation 1 with adjuvant (SP baculovirus production) | SP | 2 | Day 0 + 21 | IM | Sanofi Pasteur + GSK | Phase 3 |  | [NCT04537208](https://clinicaltrials.gov/ct2/show/NCT04537208) | [NCT04762680](https://clinicaltrials.gov/ct2/show/NCT04762680) |  | [PACTR202011523101903**](https://pactr.samrc.ac.za/TrialDisplay.aspx?TrialID=13475) |  | Ib | A |
|  |  |  |  |  |  |  |  |  |  |  | [Study Report](https://www.thelancet.com/journals/laninf/article/PIIS1473-3099(21)00147-X/fulltext) |  |  |  |  |  |  |
| 69 |  | SCB-2019 | SCB-2019 + AS03 or CpG 1018 adjuvant plus Alum adjuvant (Native like Trimeric subunit SP vaccine) | SP | 2 | Day 0 + 21 | IM | Clover Biopharmaceuticals Inc./GSK/Dynavax | Phase 2/3 | [NCT04405908](https://clinicaltrials.gov/ct2/show/NCT04405908?term=clover&cond=covid-19&draw=2&rank=1) |  |  | [NCT04672395](https://clinicaltrials.gov/ct2/show/NCT04672395) |  |  | Ib | A |
|  |  |  |  |  |  |  |  |  |  | [Study Report](https://www.thelancet.com/journals/lancet/article/PIIS0140-6736(21)00241-5/fulltext) |  |  |  |  |  |  |  |
|  |  |  |  |  |  |  |  |  |  | [Report](https://www.medrxiv.org/content/10.1101/2020.12.03.20243709v1) |  |  |  |  |  |  |  |
| 70 |  | COVAX19 | Recombinant SP + adjuvant | SP | 1 | Day 0 + 21 | IM | Vaxine Pty Ltd. | Phase 1 | [NCT04453852](https://clinicaltrials.gov/ct2/show/NCT04453852?term=vaccine&cond=covid-19&draw=5) |  |  |  |  |  |  |  |
| 71 |  | MVC-COV1901 | S-2P protein + CpG 1018 | SP | 2 | Day 0 + 28 | IM | Medigen Vaccine Biologics + Dynavax + National Institute of Allergy and Infectious Diseases (NIAID) | Phase 2 | [NCT04487210](https://clinicaltrials.gov/ct2/show/study/NCT04487210?term=vaccine&cond=covid-19&draw=7) |  | [NCT04695652](https://clinicaltrials.gov/ct2/show/NCT04695652) |  |  |  |  |  |
|  |  |  |  |  |  |  |  |  |  |  |  | [NCT04822025](https://clinicaltrials.gov/ct2/show/NCT04822025?term=vaccine&type=Intr&cond=Covid19&draw=2&rank=6) |  |  |  |  |  |
| 72 |  | FINLAY-FR1 | anti-SARS-CoV-2 Vaccine (RBD + adjuvant) | RBD | 2 | Day 0 + 28 | IM | Instituto Finlay de Vacunas | Phase 1/2 | [RPCEC00000338](https://rpcec.sld.cu/en/trials/RPCEC00000338-En) | [RPCEC00000332](https://rpcec.sld.cu/en/trials/RPCEC00000332-En) | [RPCEC00000366](https://rpcec.sld.cu/trials/RPCEC00000366-En) |  |  |  |  |  |
| 73 |  | FINLAY-FR2 | anti-SARS-CoV-2 Vaccine (RBD chemically conjugated to tetanus toxoid plus adjuvant) | RBD | 2 | Day 0 + 28 | IM | Instituto Finlay de Vacunas | Phase 3 | [RPCEC00000340](https://rpcec.sld.cu/en/trials/RPCEC00000340-En) |  | [RPCEC00000347](https://rpcec.sld.cu/en/ensayos/RPCEC00000347-Sp) |  | [RPCEC00000354](http://https/rpcec.sld.cu/en/trials/RPCEC00000354-En) |  |  |  |
| 74 |  | EpiVacCorona | EpiVacCorona vaccine based on peptide antigens for the prevention of COVID-19 | peptide antigens | 2 | Day 0 + 21 | IM | Federal Budgetary Research Institution State Research Center of Virology and Biotechnology "Vector" | Phase 3 |  | [NCT04527575](https://clinicaltrials.gov/ct2/show/NCT04527575) |  |  | [NCT04780035](https://www.clinicaltrials.gov/ct2/show/NCT04780035?term=vaccine&cond=Covid19&draw=2) |  |  |  |
| 75 |  | Recombinant SARS-CoV-2 vaccine (Sf9 Cell) | RBD (baculovirus production expressed in Sf9 cells) | RBD | 2 | Day 0 + 28 | IM | West China Hospital + Sichuan University | Phase 2 | [ChiCTR2000037518](http://www.chictr.org.cn/showprojen.aspx?proj=60581) |  | [ChiCTR2000039994](http://www.chictr.org.cn/showprojen.aspx?proj=64449) |  |  |  |  |  |
|  |  |  |  |  |  |  |  |  |  | [NCT04530656](https://clinicaltrials.gov/ct2/show/NCT04530656) |  | [NCT04640402](https://clinicaltrials.gov/ct2/show/NCT04640402) |  |  |  |  |  |
|  |  |  |  |  |  |  |  |  |  |  |  | [NCT04718467](https://clinicaltrials.gov/ct2/show/NCT04718467) |  |  |  |  |  |
| 76 |  | IMP CoVac-1 | SARS-CoV-2 HLA-DR peptides | peptide antigens | 1 | Day 0 | SC | University Hospital Tuebingen | Phase 1 | [NCT04546841](https://clinicaltrials.gov/ct2/show/NCT04546841?term=vaccine&cond=covid-19&draw=2&rank=1) |  |  |  |  |  |  |  |
| 77 |  | UB-612 | Multitope peptide based S1-RBD-protein based vaccine | RBD | 2 | Day 0 + 28 | IM | Vaxxinity | Phase 2/3 | [NCT04545749](https://clinicaltrials.gov/ct2/show/NCT04545749?cond=NCT04545749&draw=2&rank=1) |  | [NCT04773067](https://www.clinicaltrials.gov/ct2/show/NCT04773067?id=NCT04780659+OR+NCT04756830+OR+NCT04773665+OR+NCT04765436+OR+NCT04762680+OR+NCT04785144+OR+NCT04758273+OR+NCT04756323+OR+NCT04761822+OR+NCT04776317+OR+NCT04773067+OR+NCT04750343+OR+NCT04742738+OR+NCT04784767+OR+NCT04760132+OR+NCT04754594+OR+NCT04760743+OR+NCT04760730+OR+NCT04742842+OR+NCT04783311+OR+NCT04748471+OR+NCT04756271+OR+NCT04775069+OR+NCT04764422+OR+NCT04758962+OR+NCT04765384+OR+NCT04743011&draw=2&rank=8&load=cart) | [NCT04683224](https://www.clinicaltrials.gov/ct2/show/NCT04683224?id=NCT04639466+OR+NCT04655625+OR+NCT04662697+OR+NCT04683224+OR+NCT04668339+OR+NCT04674189+OR+NCT04665258+OR+NCT04646590+OR+NCT04642638+OR+NCT04656613+OR+NCT04648800+OR+NCT04649515+OR+NCT04677660+OR+NCT04668625+OR+NCT04649021+OR+NCT04649151+OR+NCT04659486+OR+NCT04664075&draw=2&rank=1&load=cart) |  |  |  |  |
| 78 |  | AdimrSC-2f | recombinant RBD +/- Aluminium | RBD | ND | ND | ND | Adimmune Corporation | Phase 1 | [NCT04522089](https://clinicaltrials.gov/ct2/show/record/NCT04522089) |  |  |  |  |  |  |  |
| 79 |  | CIGB-669 | RBD+AgnHB | RBD | 3 | Day 0 + 14 + 28 or Day 0 +28 + 56 | IN | Center for Genetic Engineering and Biotechnology (CIGB) | Phase 1/2 |  | [RPCEC00000345](https://rpcec.sld.cu/en/trials/RPCEC00000345-En) |  |  |  |  |  |  |
| 80 |  | CIGB-66 | RBD+aluminium hydroxide | RBD | 3 | Day 0 + 14 + 28 or Day 0 +28 + 56 | IM |  | Phase 3 |  | [RPCEC00000346](https://rpcec.sld.cu/en/trials/RPCEC00000346-En) |  |  | [RPCEC00000359](https://rpcec.sld.cu/trials/RPCEC00000359-En) |  |  |  |
| 81 |  | BECOV2 | containing RBD of SARS-CoV-2 with four formulations, BECOV2D, BECOV2C,BECOV2B and BECOV2A | RBD | 2 | Day 0 + 28 | IM | Biological E. Limited | Phase 1/2 |  | [CTRI/2020/11/029032](http://www.ctri.nic.in/Clinicaltrials/pmaindet2.php?trialid=48329) |  |  |  |  |  |  |
| 82 |  | Nanocovax | Recombinant Sars-CoV-2 SP, Aluminum adjuvanted | SP | 2 | Day 0 + 21 | IM | Nanogen Pharmaceutical Biotechnology | Phase 1/2 |  | [NCT04683484](https://www.clinicaltrials.gov/ct2/show/NCT04683484?term=NCT04683484&draw=2&rank=1) |  |  |  |  |  |  |
| 83 |  | S-268019 | Recombinant protein vaccine (using Baculovirus expression vector system) | peptide antigens | 2 | Day 0 + 21 | IM | Shionogi | Phase 1/2 |  | [jRCT2051200092](https://jrct.niph.go.jp/en-latest-detail/jRCT2051200092) |  |  |  |  |  |  |
| 84 |  | AKS-452 | SARS-CoV-2-RBD-Fc fusion protein | RBD | 1-2 |  | SC or IM | University Medical Center Groningen + Akston Biosciences Inc. | Phase 1/2 |  | [NCT04681092](https://clinicaltrials.gov/ct2/show/NCT04681092?term=NCT04681092&draw=2&rank=1) |  |  |  |  |  |  |
| 85 |  | COVAC-2 | COVAC-1 and COVAC-2 sub-unit vaccine (SP) + SWE adjuvant | SP | 2 | Day 0 + 28 | IM | University of Saskatchewan | Phase 1/2 |  | [NCT04702178](https://clinicaltrials.gov/ct2/show/NCT04702178) |  |  |  |  |  |  |
| 86 |  | GBP510 | Recombinant surface protein vaccine with adjuvant AS03 (aluminium hydroxide) | SP | 2 | Day 0 + 28 | IM | SK Bioscience Co., Ltd. and CEPI | Phase 1/2 |  | [NCT04742738](https://clinicaltrials.gov/ct2/show/NCT04742738) |  |  |  |  |  |  |
|  |  |  |  |  |  |  |  |  |  |  | [NCT04750343](https://clinicaltrials.gov/ct2/show/NCT04750343) |  |  |  |  |  |  |
| 87 |  | Razi Cov Pars | Recombinant SP vaccine | SP | 3 | Day 0 + 21 +51 | IM and IN | Razi Vaccine and Serum Research Institute | Phase 2 | [IRCT20201214049709N1](https://www.irct.ir/trial/52975) |  | [IRCT20201214049709N2](https://en.irct.ir/trial/55238) |  |  |  |  |  |
| 88 |  | SARS-CoV-2 Sclamp vaccine | MF59 adjuvanted SARS-CoV-2 Sclamp vaccine | SP | 2 | Day 0 + 28 | IM | The University of Queensland | Phase 1 | [NCT04495933](https://clinicaltrials.gov/ct2/show/NCT04495933) |  |  |  |  |  | Ib | A |
|  |  |  |  |  |  |  |  |  |  | [Study Report](https://papers.ssrn.com/sol3/papers.cfm?abstract_id=3769210) |  |  |  |  |  |  |  |
|  |  |  |  |  |  |  |  |  |  | [Study Report](https://www.thelancet.com/journals/laninf/article/PIIS1473-3099(21)00200-0/fulltext) |  |  |  |  |  |  |  |
| 89 |  | NBP2001 | SK SARS-CoV-2 recombinant surface antigen protein subunit + adjuvanted with alum | SP | 2 | Day 0 + 28 | IM | SK Bioscience Co., Ltd. | Phase 1 | [NCT04760743](https://clinicaltrials.gov/ct2/show/NCT04760743) |  |  |  |  |  |  |  |
| 90 |  | SpFN | Spike ferritin nanoparticle; uses SPs with a liposomal formulation QS21 (ALFQ) adjuvant | SP | 2-3 | Day 0 + 28 + 180 | IM | Walter Reed Army Institute of Research (WRAIR) | Phase 1 | [NCT04784767](https://clinicaltrials.gov/ct2/show/NCT04784767) |  |  |  |  |  |  |  |
| 91 |  | EuCorVac-19 | A SP using the recombinant protein technology and with an adjuvant. | SP | 2 | Day 0 + 21 | IM | POP Biotechnologies and EuBiologics Co.,Ltd | Phase 1/2 |  | [NCT04783311](https://www.clinicaltrials.gov/ct2/show/NCT04783311?id=NCT04780659+OR+NCT04756830+OR+NCT04773665+OR+NCT04765436+OR+NCT04762680+OR+NCT04785144+OR+NCT04758273+OR+NCT04756323+OR+NCT04761822+OR+NCT04776317+OR+NCT04773067+OR+NCT04750343+OR+NCT04742738+OR+NCT04784767+OR+NCT04760132+OR+NCT04754594+OR+NCT04760743+OR+NCT04760730+OR+NCT04742842+OR+NCT04783311+OR+NCT04748471+OR+NCT04756271+OR+NCT04775069+OR+NCT04764422+OR+NCT04758962+OR+NCT04765384+OR+NCT04743011&draw=2&rank=3&load=cart) |  |  |  |  |  |  |
| 92 |  | ReCOV | Recombinant two-component spike and RBD protein COVID-19 vaccine (CHO cell). | SP/RBD | 2 | Day 0 + 21 | IM | Jiangsu Rec-Biotechnology | Phase 1 | [NCT04818801](https://clinicaltrials.gov/ct2/show/NCT04818801) |  |  |  |  |  |  |  |
| 93 |  | V-01 | Recombinant SARS-CoV-2 Fusion Protein Vaccine | SP | 2 | Day 0 + 21 | IM | Guangdong Provincial Center for Disease Control and Prevention/Gaozhou Center for Disease Control and Prevention | Phase 2 | [ChiCTR2100045108](http://www.chictr.org.cn/showproj.aspx?proj=124140) |  | [ChiCTR2100045107](http://www.chictr.org.cn/showproj.aspx?proj=124702) |  |  |  |  |  |
| 94 |  | Recombinant SARS-CoV-2 Vaccine (CHO cell) | Recombinant SARS-CoV-2 Vaccine (CHO cell) | SP | 2 | Day 0 | IM | National Vaccine and Serum Institute, China | Phase 1/2 |  | [NCT04869592](https://clinicaltrials.gov/ct2/show/NCT04869592?term=NCT04869592&draw=2&rank=1) |  |  |  |  |  |  |
| 95 |  | CoVepiT | SARS-CoV-2 multi-target peptide vaccine (targeting Spike, M, N, and several non-structural proteins) | SP/N/M/Non-structural proteins | 1-2 | Day 0 +/- 21 | SC | OSE Immunotherapeutics | Phase 1 | [NCT04885361](https://www.clinicaltrials.gov/ct2/show/NCT04885361?term=NCT04885361&draw=2&rank=1) |  |  |  |  |  |  |  |
| 96 | Virus like particle | SARS-CoV-2 HBsAg VLP vaccine | RBD SARS-CoV-2 HBsAg VLP vaccine | RBD | 2 | Day 0 + 28 | IM | Serum Institute of India + Accelagen Pty + SpyBiotech | Phase 1/2 |  | [ACTRN12620000817943](https://anzctr.org.au/Trial/Registration/TrialReview.aspx?id=380145&isReview=true) |  |  |  |  |  |  |
|  |  |  |  |  |  |  |  |  |  |  | [ACTRN12620001308987](https://anzctr.org.au/Trial/Registration/TrialReview.aspx?ACTRN=12620001308987) |  |  |  |  |  |  |
| 97 |  | CoVLP | Coronavirus-Like Particle COVID-19 | SP | 2 | Day 0 + 21 | IM | Medicago Inc. | Phase 2/3 | [NCT04450004](https://clinicaltrials.gov/ct2/show/NCT04450004) |  | [NCT04662697](https://www.clinicaltrials.gov/ct2/show/NCT04662697?term=vaccination&cond=covid&draw=1&rank=86) | [NCT04636697](https://clinicaltrials.gov/ct2/show/NCT04636697) |  |  |  |  |
|  |  |  |  |  |  |  |  |  |  | [Study Report](https://www.medrxiv.org/content/10.1101/2020.11.04.20226282v1) |  |  |  |  |  |  |  |
| 98 |  | VBI-2902a | An enveloped virus-like particle (eVLP) of SARS-CoV-2 SP and aluminum phosphate adjuvant. | SP | 2 | Day 0 + 28 | IM | VBI Vaccines Inc. | Phase 1/2 |  | [NCT04773665](https://clinicaltrials.gov/ct2/show/NCT04773665) |  |  |  |  |  |  |
| 99 |  | SARS-CoV-2 VLP Vaccine | VLP adjuvanted vaccine | RBD | 2 | Day 0 | SC | The Scientific and Technological Research Council of Turkey | Phase 1 | [NCT04818281](https://clinicaltrials.gov/ct2/show/NCT04818281?cond=NCT04818281&draw=2&rank=1) |  |  |  |  |  |  |  |
| 100 |  | COUGH-1 | ABNCoV2 capsid virus-like particle (cVLP) +/- adjuvant MF59 | SP | 2 | Day 0 + 28 | IM | Radboud University | Phase 1 | [NCT04839146](https://clinicaltrials.gov/ct2/show/NCT04839146) |  |  |  |  |  |  |  |

**Data source:** <https://biorender.com/covid-vaccine-tracker;> <https://www.who.int/who-documents-detail/draft-landscape-of-covid-19-candidate-vaccines>

**Abbreviation: rAd5:** recombinant adenovirus 5; **rAd26:** recombinant adenovirus 26; **SP:** spike protein; **RBD:** receptor-binding-domain; **ChAdOx1-S:** attenuated resulting in infections in chimpanzees; **LNPs:** lipid-based nano-paticles
